# Convergence and divergence of songs suggests ongoing, but annually variable, mixing of humpback whale populations throughout the North Pacific

**DOI:** 10.1038/s41598-019-42233-7

**Published:** 2019-05-07

**Authors:** James D. Darling, Jo Marie V. Acebes, Oscar Frey, R. Jorge Urbán, Manami Yamaguchi

**Affiliations:** 1 Whale Trust, Makawao, Hawaii 96768 United States of America; 2 BALYENA.ORG, Barangay Pangdan, 6308, Philippines and National Museum of the Philippines, Zoology Division, Manila, 1000 Philippines; 3 Deep Blue Conservancy, Puerto Vallarta, Jalisco, CP 48328 Mexico; 4 Universidad Autonoma de Baja California Sur, Departamento de Ciencias Marinas y Costeras, La Paz, Mexico; 5 Ogasawara Club, Komagari, Chichi-jima, Ogasawara, Tokyo, Japan

**Keywords:** Behavioural ecology, Animal behaviour, Animal migration, Behavioural ecology, Marine biology

## Abstract

All humpback whale (*Megaptera novaeangliae*) males in a population sing fundamentally the same version of a complex, progressively changing, series of sounds at any one time – the song. The purpose of this study was to describe the relationship of humpback whale populations across the North Pacific based on song composition. Songs were collected from Philippines, Japan, Hawaii and Mexico in 2011, 2012 and 2013. The presence and proportion of 11 phrase types were compared within and between populations to investigate song similarity and change. Results included: shared song phrases across the North Pacific; variable, temporary, regional song differences; varying rate of song change; and distance a factor, but not predictor in degree of similarity. Shared phrases indicate ongoing mixing of populations throughout the North Pacific. Year to year differences in degree of similarity suggest variability in these interactions. Songs appear to diverge as populations split up and converge when they amalgamate. Song studies complicate current US management policy designating four distinct populations in the North Pacific. North and South Pacific humpback whale population structure may be comparable, although song dynamics may be different. The fluidity of song composition suggests it provides acoustic definition or identity to changing associations of whales.

## Introduction

Humpback whale (*Megaptera novaeangliae*) males repeat loud, long (5–20 min), complex sequences of sounds known as song^1,2^. Singing is largely but not exclusively seasonal, its occurrence increasing in fall feeding grounds, peaking during winter migrations and assembles, and decreasing in the spring^3–8^. Its function is not known but is generally presumed to play a role in breeding activities^9–14^.

The composition of the song, that is, the presence and arrangement of specific sound units, progressively (or abruptly) changes, yet all the singers in one population sing fundamentally the same version at any one time^3,4^. How exactly this is accomplished and how changes are incorporated throughout the population is not clear, but apparently involves ongoing mutual melding and/or one-way adoption of songs of neighboring singers, a form of cultural transmission^4,15,16^. Regardless of the mechanism, this dynamic works to identify whales recently associated, at least acoustically, with each other. Similar composition of the song indicates some degree of association between whales; different composition indicates no recent association of these animals.

The practical application of these song characteristics to define humpback whale populations has been proposed since the late 1970s with the first comparisons of songs between Mexico and Hawaii^17,18^. Since then, further song comparisons in the North Pacific^19–23^ and worldwide^24–28^ have, at least, inferred that the similarity or difference in songs is a reflection of the degree of population mixing.

However, to date the song has not been widely recognized as a means of population definition in humpback whale management. In fact, a recent regulatory action under the Endangered Species Act  by U.S. National Oceanic and Atmospheric Association (NOAA)/National Marine Fisheries Service (NMFS) ignored songs entirely, with the declaration of population units based on “genetic data, tagging and photographic-ID data, demographic information, geographic barriers and stranding data”^29^. This action divided the North Pacific population into four “Distinct Population Segments” (DPSs) based on winter assemblies, regardless of multiple studies indicating similarity of song^17,18,21–23^.

Beyond potential human use of song to distinguish whale populations is the question of whether or not it serves a similar function for the whales, that is, the identification of recent associates^11^. If, indeed, the song functions as a signal of association on a population level, could the same song dynamic work on a smaller scale and provide an identity to smaller groups, or even individuals?

Both management-use issues and the study of song function come down to the same question: does the degree of song similarity correlate with degree and/or immediacy of interaction? Or, as a hypothesis: on any scale, whales that are more associated – that is, engage in more interactions – should have a more similar song than those that are lesser associated.

This current study expands on an initial investigation into this question undertaken in 2006 with a “snapshot” comparison from three locations over a single season^23^. This earlier study compared locations in the Philippines, Japan and Hawaii. Consistent with the hypothesis, the songs of the two geographically closer locations (Philippines and Japan) were more similar than when compared with songs from the more distant Hawaii. The finer the song characteristics examined, the more definable any temporarily isolated population was. However, overshadowing these real but relatively small differences apparent over this single season was the marked similarity of songs from Hawaii and Asia, and this, combined with past Hawaii-Mexico studies^18–21^, suggested mixing of humpback whales throughout the Pacific. While a notable ocean-wide similarity in songs existed, smaller scale differences apparently reflected the degree of separation of populations.

The purpose of the current study then was two-fold. First, to further investigate the interaction and identity of humpback whale populations in the North Pacific based on song composition; and second, to examine how these results might contribute to investigation of the function of the song.

## Methods

### Study locations

**Figure 1:**
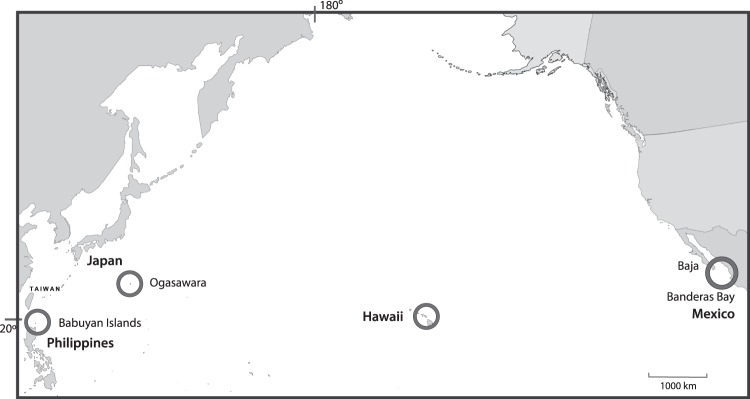
The study areas in Philippines, Japan, Hawaii and Mexico where humpback whale song recordings were collected in 2011, 2012 and 2013. The approximate distances between the study locations are: Babuyan Islands to Ogasawara, 2,300 km; Ogasawara to Hawaii, 6,200 km; Babuyan Islands to Hawaii, 8,400 km; Hawaii to Mexico, 4,800 km; Ogasawara to Mexico 10,500 km, Babuyan Islands to Mexico, 12,500 km.

The study locations were in four regions as shown in Fig. 1: in the northern Philippines, the Babuyan Islands (19°01′N, 121°38′E); in Japan, the Ogasawara Islands 960 km southeast of Tokyo (27°05′N, 142°04′E); in the Hawaiian Islands, Maui (20°45′N, 156°40′W); and in Mexico, two locations: S.E. Baja Peninsula (23°50′N, 109°28′W) and Banderas Bay on the adjacent mainland (20°38′N105°24′W). In this analysis, the two Mexican locations due to closeness (500 km apart) were treated as one^30^. The distances between the locations and the humpback whale populations compared are provided in Fig. 1.

### Song samples

The objective was to record multiple singers, in all locations, over as much of the winter season as possible in each of these three study years. The total sample from each region in each year varied with field circumstances, including duration of study, weather, equipment issues and whale presence. Samples were collected directly from small (7–10 m) craft with dipping hydrophones. A singer was located acoustically, and often visually; the boat maneuvered until the signal was strong and the song was recorded.

Recording equipment varied between locations, but all were professional quality digital recorders and hydrophones as follows: Philippines, Zoom H4N recorder with DolphinEAR Pro hydrophone; Japan, in 2011–12, SONY DAT Walkman TCD-D50 and in 2013, SONY Linear PCM Recorder PCM-D50, with HTI-96-min hydrophone; Hawaii, Marantz 620 with HTI-9-min hydrophone; Mexico: Baja, Marantz PMD 660 with HTI-96-min hydrophone, and Banderas Bay, in 2011, Sony Hi-MD recorder (MZ-M200) with Offshore Acoustics omnidirectional hydrophone, in 2012–13, a Fostex FR2 Field recorder with Cetacean Research Technology directional hydrophone (C304). All songs were recorded in wav format with a sampling rate of 44.1 kHz or higher, with the exception of two of the four 2011 Philippine songs recorded as MP3 files (converted to wav format for analysis).

All recordings of at least 20 minutes duration, or that clearly included one full song cycle (one sequence of all the phrases the singer is currently using before repeating) were graded for clarity. The quality was assessed subjectively by the principle investigator (JD) – the only criteria being that all components of the song were clear, both aurally and visually on spectrograph. The lowest grade used in analysis was ‘C+’, which signified recordings with ‘moderate background noise (anthropogenic or other whales) but with all sound units recognizable. The majority of recordings were ‘A’ quality with a strong signal and quiet background. The song samples used in this analysis, including combined length of recordings, number of singers, and date span of recordings, are summarized in Table 1.

**Table 1:** Sample of songs, 2011–2013, used in analysis. Listed per year: the total sample time (seconds), the number of singers the sample was obtained from, and the span of dates over which recordings were made in each location.

	Philippines	Japan	Hawaii	Mexico
**2011**				
Total sample (s)	8200	11130	13995	5530
No. singers	4	4	9	8
Date span	3 Apr.–18 Apr.	17 Apr.–30 Apr.	23 Jan.–10 Apr.	8 Jan.–13 Mar.
**2012**				
Total sample (s)	26395	16095	22685	14305
No. Singers	13	7	18	8
Date span	6 Mar.–16 Apr.	7 Mar.–21 Apr.	14 Dec.–7 Apr.	8 Feb. −16 Mar.
**2013**				
Total sample (s)	10740	20325	17170	7885
No. Singers	2	11	16	13
Date Span	12 Mar.–13 Mar.	30 Jan.–21 Apr.	7 Jan.–11 Apr.	15 Jan.–19 Mar.

Recordings from each singer ranged from the length of one song cycle (the shortest at 9 min 20 s) to an hour or longer and over several song cycles. Some recordings also included partial songs when the recorder was turned on or off mid-song. In this analysis, *all* recordings that met the criteria of length and quality were included, both full song and fragments. The reasons for this were: (1) it was not always obvious in recordings where the ‘beginning’ and ‘end’ of the song cycle was. Some whales surfaced and dove mid-song, that is between the most common first and last phrases; and, in fact, some did not include first and last phrases in a cycle; and, (2) one purpose of this analysis was to obtain as full a representation as possible of all phrase types in a population, including those which may have occurred even once in a fragment of a song. Analyses within this study determined if a pattern was common or rare.

### Analysis and comparison

**Figure 2:**
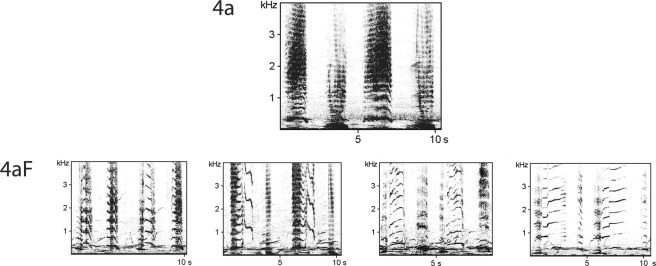
Examples of unit flourishes (lower row) in Phrase 4a (above). The phrase consists of two units; uttered twice in each box. The range of flourishes were clumped under one designation, 4aF, in this analysis (FFT 512, 50% overlap, Hamming window; sampling rate 44.1 kHz).

This analysis is based on the hierarchical song structure (sound units arranged as phrases repeated as a theme) with terms first described in 1971^1^ and used by most authors since^3,4,14–16,18,20–23,25,26,31^. Terms are summarized in Table 2. The only terms not used in previous studies were *parent phrase* and *unit flourish* as defined below (Table 2), and in the latter case with examples provided in Fig. 2 and Supplementary Material Fig. S2.

**Table 2:** Definition of terms used in this study.

Unit	Single, distinguishable continual sound (as audible to human ear).
Phrase	Sound units in a specific, repetitive arrangement, usually (but not necessarily) composed of several different sounds.
Parent phrase	The initial phrase to which small changes may occur, leading to a variant. As change is a continuing process, this designation is relative to the time period analyzed.
Phrase variant	A slightly different version of a phrase due to consistent, ‘minor’ modification of the unit arrangement (within the overall pattern of the phrase). For example, separate units in a phrase join, one unit is dropped or one unit split into two. Variants often alternated back and forth with other variants of the same phrase.
Unit flourish	One or more units in a phrase modified in frequency and duration that complicate or enhance the unit (think improvisational extensions of single notes in Jimi Hendrix’ *Star Spangled Banner*). The composition of the phrase does not change, but rather the expression of a particular sound. There may be several different flourishes of the same sound unit (Fig. 2). Often a singer alternated between ‘simple’ and ‘flourished’ units in one series of the same phrase. One individual could produce several different flourishes; different individuals could produce the same flourish.
Theme	Segment of a song that comprises all the repetitions of the same phrase (including its variants).
Song cycle	One sequence of all the phrases a singer is currently using, before repeating.
Transition	A short (typically seconds long) segment of the song where a whale shifts from one theme to the next and may mix sound units from both.
Hybrid phrase	A distinctive phrase composed of units from two other phrases in the song. A hybrid phrase typically recurs in a sample, is of longer duration than a transition, and is composed of units not from successive phrases.

Recordings were examined aurally and with spectrograph (using Avisoft-SASLab Pro and Raven Pro 1.5 software) to identify and define phrase types; examples provided in Supplementary Fig. S1. This identification was purposefully done by one person (JD) to ensure consistency. Each phrase type was given an alphanumeric label reflecting its distinctiveness, and given a color for ease of recognition. Spectrograph illustration, color designation, unit arrangement, and acoustic parameters for each phrase and phrase variant, including a unit flourish example, are provided in Supplementary Fig. S2 and Supplementary Table S2. Audio files matching the spectrographs in Supplementary Fig. S2 are available on request.

Phrases were the primary unit of comparison between locations and years. However, in this 2011–2013 sample of songs one phrase was more complex than the others, and was divided into four sub-patterns described in Results. The presence and proportion of these sub-patterns were also used as comparison points.

The analysis consisted of determining if a specific phrase type (phrase, phrase variant or unit flourish) was present in a recording and, if so, how common (percentage of sample) it was. For the latter, all individual song recordings in each location/year were combined as a ‘population sample’. In this total sample the amount of time (seconds) dedicated to each Phrase Type was determined, and its proportion stated as a percent. (For example in Hawaii in 2013, in 16 singers, Phrase 3 was uttered for a total of 1200 seconds in the 171,170 second song sample, hence 7% of the ‘population song’. See Supplementary Material Table S1 for a more detailed description and calculation of this example.) This process ensured that the full repertoire of phrase types in the population was included, since all types are not included in all songs or even in all song sessions of one individual. This study compared populations per year, not individuals nor, with one exception noted below, shorter time spans.

In the vast majority of songs used in this study, the phrases (and variants) were highly distinctive points of comparison. However, continual, gradual song change over the study period, and individual singer variation across a sample required decisions on: (1) the splitting or clumping of relatively minor variations in phrase types; (2) defining when one phrase type changed enough to be called a different phrase type; and (3) resolution of sound patterns inconsistent or exceptional to the norm. These decisions were made after the initial assessment of the data, and then applied consistently throughout the analyses. More detail on these decisions is included in the Supplementary Methods − Analysis Decisions, and in Supplementary Table S2.

Results of the broader analysis led to two other small explorations of the data. The 2013 Japan song sample was divided into early and late season and these halves compared to more accurately illustrate song changes within this one season.

To provide some context for the  Mexico  2011 song, songs from Mexico and Hawaii in 2010, the year prior to this study, were examined.

### Statistical treatment

With the comparisons of representative songs from four locations over three years, (that is, with *n* values of 4 and 3), statistical analyses were limited. However, once the presence and percent occurrence of each phrase type was determined, Pearson’s Correlation Coefficient was applied between song samples providing an index of similarity between years in one location, and between the four locations in each year. Additionally, regression analyses provided a measure of degree of song similarity (using simple presence or absence of a  phrase type) versus geographical distance of separation.

### Animal experiments

There was no animal experimentation in this study. All data were the result of passive sound recording at sea with no impact on the animals. All necessary Federal and State permits to record in the vicinity of the animals were obtained.

## Results

### Phrase types

In the full three-year, four-location song sample across the North Pacific, a total of 11 phrase types were used as comparison points between the representative song for each year/location. These included eight phrases (1, 2, 3, 4, 5, 6, 7, 8) as well as finer divisions of the more complex Phrase 4. Phrase 4 was divided into the parent (4a) and two variants (4b, 4c). The parent phrase 4a was further divided into ‘simple’ (4a) versus ‘flourish’ (4aF) versions. (See Supplementary Fig. S2 and Supplementary Table S1.)

For the sake of simplicity, when the term *phrases* is used in the text it encompasses *all* of the sound patterns used as comparison points referred to above: the phrases, phrase variants and flourishes.

### Presence of phrases

**Figure 3:**
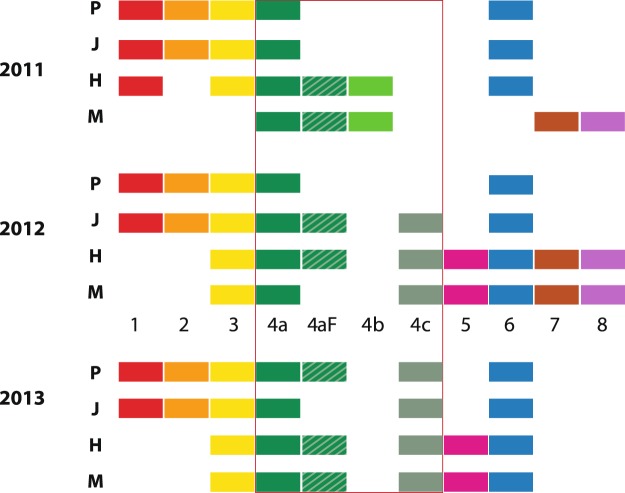
The presence of specific phrases in the different populations: Philippines (P), Japan (J), Hawaii (H) and Mexico (M) each year. Each phrase is indicated by a different alphanumeric label (1–8) and color. Phrases 1–6 are in the general order in which they were sung in each sample; 7–8 less predictable. Phrase variants are in shades of green; the unit flourish type 4aF is represented by a stippled pattern over the 4a green. The phrase types that the colors represent are detailed in Supplementary Fig. S2 and Supplementary Table S2.

**Figure 4:**
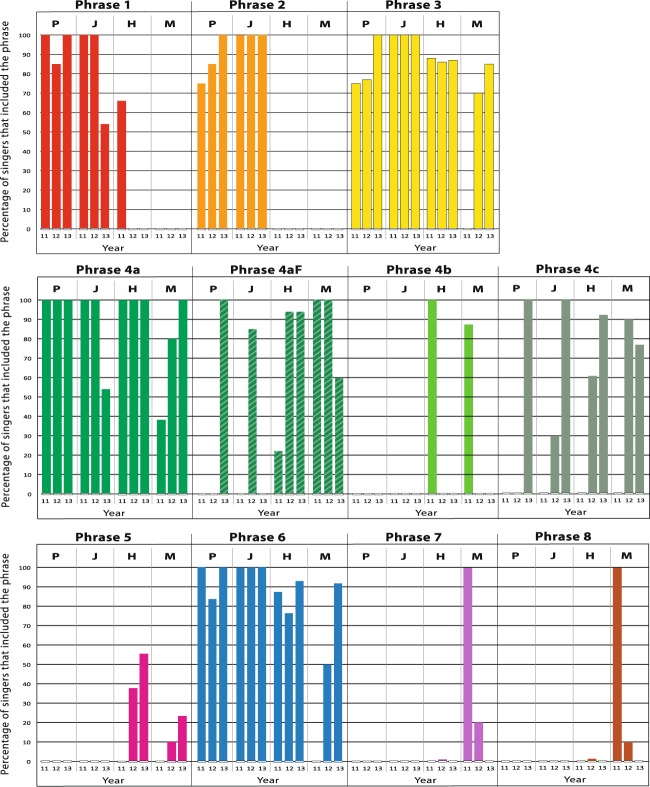
Percentage of singers in each location/year, which included the various phrases in their song.

This comparison asked simply, was a specific phrase found, even once, in a population in a season? The result is in Fig. 3. The actual percentage of singers (from each location and year) that included the specific phrases is presented in Fig. 4. This ranged widely, from some phrases present in 100% of songs in all years, to others present in 10% or just one song in one year.

During the three years of this study some phrases were shared between populations across the North Pacific while others were more exclusive to opposite sides of the ocean. Five phrases (3, 4a, 4aF, 4c, 6) were identified in all four locations across the span of the Pacific; seven phrases were found in three of the four; and all 11 found in two of the four locations at some point within the three-year period as shown in Table 3. There were no phrase types unique to a single study population for the three-year period (Fig. 3, Table 3). On the other hand, two phrases (Phrase 1, 2), found in all three years in the two Asia populations, were not identified in the most geographically distant Mexico song. Phrase 1 was documented in Hawaii but only in 2011. In contrast, Phrases 7 and 8, present in two of three years in Mexico, were not found in the most distant Asia songs, but were identified once in Hawaii in 2012. Phrase Variant 4b and Phrase 5 were only found in Mexico and Hawaii samples, not in Asia.

**Table 3:** Presence of phrases (including variants and flourish) in each location/year.

Phrase	Presence (Location/Year)													
	Philippines			Japan			Hawaii			Mexico			Preval.	No. Loc.
Year:	11	12	13	11	12	13	11	12	13	11	12	13		.
1	X	X	X	X	X	X	X						7	3/4
2	X	X	X	X	X	X							6	2/4
3	X	X	X	X	X	X	X	X	X		X	X	11	4/4
4a	X	X	X	X	X	X	X	X	X	X	X	X	12	4/4
4aF			X		X		X	X	X	X	X	X	8	4/4
4b							X			x			2	2/4
4c			X		X	X		X	X		X	X	7	4/4
5								X	X		X	X	4	2/4
6	X	X	X	X	X	X	X	X	X		X	X	11	4/4
7								X		X	X		3	2/4
8								X		X	X		3	2/4
Total														
11	5	5	7	5	7	6	6	8	6	5	8	6		

Lowest row: total number of phrase types found in the song in each location/year. Preval. (prevalence) column: the number of location/year categories the sound pattern appeared in. (max. 12 possibilities, i.e. 4 locations, 3 years). No. Loc. (number of locations) column: of the 4 possible geographic locations, how many the phrase types appeared in at some point during the 3-year period. For example, Phrase 1 appeared in 7 of a possible 12 opportunities and in 3 of 4 possible locations.

There were, within the three-year span, year-to-year differences in both total phrases in a population (as phrase types appeared and disappeared), and in the percentage of these shared between the North Pacific populations (Table 3). The first year of the study, 2011, was the year of least similarity across the North Pacific: only one (Phrase 4a) of a total nine phrases (11%) was shared amongst all four populations. In 2013, the year of most similarity, four (Phrases 3, 4, 5, 6,) of a total of eight phrases (50%) were shared in all four populations. The shared percentages were higher for any three locations, or any two. For example, in 2012 and 2013, two locations, Mexico and Hawaii, shared 100% of phrases. The songs’ prominent Phrase 4, with its potential variants and flourish, was the one common phrase in the least common year (2011), as well as being the most common phrase throughout the entire study sample (Figs 3, 4).

### Proportional phrase representation

**Figure 5:**
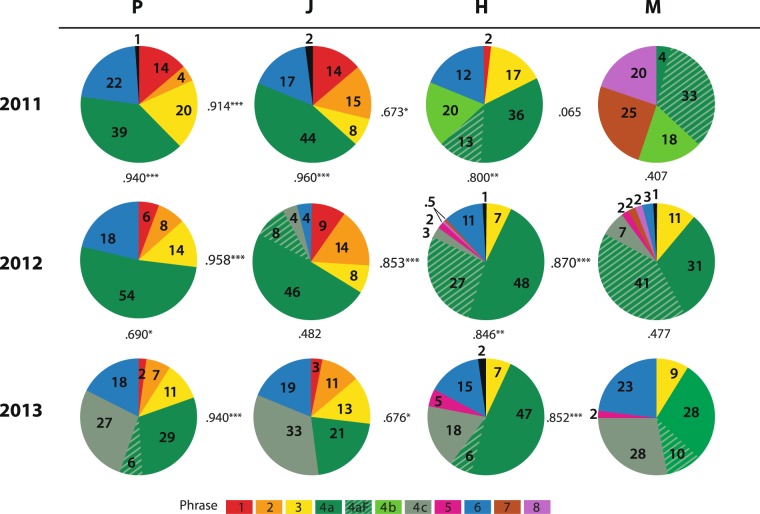
Similarity and difference of songs per year and location, Philippines (P), Japan (J), Hawaii (H) and Mexico (M) for the three years, 2011–2013. The percentage of song dedicated to each phrase is given within the corresponding color, and in the general order sung. In the columns, the song for one location is compared over three years; in the rows, the songs in all four locations compared over any one year. Pearson’s Correlation Coefficients comparing all the location and year samples with each other are provided as Supplementary Table S3. Several of these values between the years or locations are included here (significance, 0.001 level***, 0.01 level**, 0.05 level* (two-tailed)).

Beyond simple presence there was a wide range in the proportion of a song cycle dedicated to a specific phrase. This analysis determined what percentage of the song was dedicated to each of the phrase types: 1, 2, 3, 4a, 4aF, 4b, 4c, 5, 6, 7, 8. The results are illustrated in the pie charts in Fig. 5.

#### Phrase use unequal, and changing

The percentage of song cycle dedicated to any one phrase ranged from less than 1% to close to 80% (clumping variants), with some consistency in this value per phrase across all populations in a year. For example, Theme 4 (all variants combined) represented the largest percentage of the song in all locations in all years, and comprised more than 50% of the song sample in 10 of 12 possible combinations of locations/years (Fig. 5). When all samples in all locations were combined, the mean proportion of Theme 4 (green) was 61% of the song cycle; the next most prevalent themes, in all samples combined, were Theme 6 (blue) at 13%, and Theme 3 (yellow) at 10%, with all other themes below 10%.

Over the three years of this study, the proportion of the song the whales spent on a specific phrase changed. For example, Phrase 1 (red) dropped from, in 2011, 14% percent of the song in both Philippines and Japan and 2% in Hawaii; to, in 2012, 6%, 9% and 0% respectively; and in 2013, even lower to 2%, 3% and 0%. A more rapid decrease and disappearance of full phrases occurred in Mexico with Themes 7 and 8 occupying 20% and 25% of song in 2011; by 2012, this was down to 2% and 2%, and by 2013, both were at 0%. Other phrases maintained their presence and proportion over the three years (e.g., Phrase 3, yellow), while new phrases appeared during the study period, such as Phrase 5 (pink), which progressed from 0% in 2011, to 2% in 2012, to 5% in 2013.

#### Pace of song change variable

Within the three years the amount of song change (as defined by proportional representation of phrases) from year to year was not a constant within a population. For example, the Philippines’ song showed virtually no change from 2011 to 2012 with a high correlation (r = 0.940, p < 0.0005), and a greater change from 2012 to 2013 with weaker (r = 0.690, p = 0.013) between these years (Fig. 5). The 2011 and 2012 were relatively simple, five-phrase songs (all phrases with no variants and no flourish), with the only changes from year to year being minor shifts in the proportion of song spent on different phrases. Between 2012 and 2013 a greater degree of change occurred with the emergence of two new patterns within Phrase 4: 4aF and 4c, not present in 2011. By 2013, the song had become more complex with a total 7 phrase types rather than 5.

A similar pattern occurred in the Japan song with 2011 and 2012 highly correlated (r = 0.960, p < 0.0005) and 2012 to 2013 with a much lower correlation (r = 0.482, p = 0.112) (Fig. 5). (Further examination of this process in Japan is addressed later in this paper). The Philippines’ and Japan songs were highly correlated each year (as well as in an earlier 2006 song comparison^23^) and remained so with the pace of change increasing similarly in both populations.

While the Asia song’s rate of change varied year to year, Hawaii and Mexico’s were more consistent, yet different. In Hawaii, the song correlations suggested a slow and steady change: from 2011 to 2012 (r = 0.800, p = 0.002) and from 2012 to 2013 songs (r = 0.846, p = 0.001). Over the three years from 2011 to 2013, Hawaii song changed least of the 4 locations (r = 0.714, p = 0.009). In contrast, the Mexico song showed a substantial change from 2011 to 2012 (correlation: r = 0.407, p = 0.189) and again, from 2012 to 2013 (r = 0.477, p = 0.117). The Mexico song changed most in the three years with an inverse correlation value (r = −0.255, p = 0.423) between 2011 and 2013 songs. Over the three years, the Asia song changed more than Hawaii and less than Mexico (2011 to 2013: Philippines, r = 0.644, p = 0.024 and Japan, r = 0.454, p = 0.139) but, as discussed above, it changed unsteadily, with most of that change coming in the last year.

In the three years of this study, the Mexico population was most ‘acoustically dynamic’, with correlations year to year of approximately 0.4 and 0.4, or roughly 60% of song change per year. In comparison, in Hawaii, the year-to-year correlations were 0.8 and 0.8 or 20% change per year. Asia, as described above, showed variability in this regard, ranging from a less than 10% to near 50% song change per year.

### Year to year variability in how similar songs were across the North Pacific

Over the course of this study, the degree of similarity of songs between locations was not consistent year to year, and varied substantially (Fig. 5). At their extremes, song correlation values between the most distant populations on either side of the North Pacific (Asia and Mexico) ranged all the way from inverse (r = −0.398, p = 0.200) in 2011, to highly correlated (r = 0.964, p < 0.0005) in 2013.

In 2011 (Fig. 5 top row), from West to East, the Philippine and Japan songs were highly correlated (r = 0.914, p < 0.0005) and Hawaii song was moderately correlated with the Asia song (with Japan r = 0.673, p = 0.016, with Philippines r = 0.743, p = 0.006). The difference between Asia and Hawaii song this year was that Asia included five basic phrases, whereas Hawaii subdivided Phrase 4 into 4b and 4aF, and did not include Phrase 2 common in Asia. In 2011, the Mexico song showed least correlation with other songs in this study. The Mexico song was ‘relatively’ more similar with Hawaii with this very weak correlation value (r = 0.065, p = 0.842) as compared to the Asia song to which it was inversely correlated. In 2011, Mexico and Asia shared one phrase in nine, and Mexico and Hawaii shared three of eight.

In 2012 (Fig. 5 middle row) again there was a high correlation between Philippines’ and Japan songs (r = 0.958, p < 0.0005), although the latter included an appearance of Phrase 4 variants not present in the Philippines. The Asia-Hawaii correlation was high (Philippines r = 0.809, p = 0.001, and Japan r = 0.853, p < 0.0005) even with Hawaii missing two phrases (Phrase 1 and 2) present in the Asia song. The most notable change in 2012 was that the Mexico song had changed to align more with Hawaii (r = 0.870, p < 0.0005 rather than r = 0.065, p = 0.842 the previous year) and, in turn, the rest of the Pacific (r = 0.560, p = 0.058 with Japan, r = 0.451, p = 0.141 with Philippines.). This greater similarity over the previous year resulted from smaller percentages in the Mexico song (slivers of pie chart) of three phrases not in Asia, and a smaller proportion in the Asia song of two phrases not in Mexico.

 In 2013 (Fig. 5 bottom row), the high correlation (r = 0.940, p < 0.0005) between Philippines and Japan remained, however the correlation between Philippines and Mexico, the locations furthest  apart, across the span of the North Pacific, was also high (r = 0.964, p < 0.0005): With correlations of Philippines and Hawaii (r = 0.869, p < 0.0005), Japan and Hawaii (r = 0.676, p = 0.016), Mexico and Hawaii (r = 0.852, p < 0.0005) and Japan and Mexico (r = 0.879, p < 0.0005), 2013 was the year of greatest song similarity between all the North Pacific populations.

### Collective change across the Pacific over time

In two of the three years, a population’s song had a higher correlation with current songs of the other populations in the North Pacific than to its own song from the previous year – the exception being 2011 (Fig. 5). For example, the 2013 Philippine song was more similar to the 2013 Hawaii song (r = 0.869, p < 0.0005), than to the 2012 Philippines song (r = 0.690, p = 0.013). The 2013 Mexico song was more similar to songs from all other locations in 2013 (Hawaii, r = 0.852, p < 0.005; Japan r = 0.879, p < 0.0005; Philippines r = 0.964, p < 0.0005) than to its own song in 2012. (r = 0.477, p = 0.117). The Mexico song in 2012 was more similar to Hawaii in 2012 (r = 0.870, p < 0.0005) than to its own song in 2011 (r = 0.407, p = 0.189). The 2011 exception included the unusually different Mexico song that year, which was more similar to Mexico in the following year 2012 than to other songs across the North Pacific in the same year, 2011.

### Convergence of song composition across the North Pacific over the three years

The song became progressively more similar across the North Pacific over the three years of this study in both the number of shared phrase types and strength of correlations. As stated earlier, in 2011, just 1 of 9 phrase types (11%) was shared across the entire North Pacific, in 2012, 3 of 8 (30%) phrase types were shared, and in 2013, 4 of 8 (50%) phrase types shared. Similarly, correlations between samples from the most distant populations progressed from inverse in 2011 (Philippines and Mexico: r = −0.398, p = 0.200; Japan and Mexico: r = −0.377, p = 0.228) to somewhat correlated in 2012 (Philippines and Mexico: r = 0.451, p = 0.141; Japan and Mexico: r = 0.560, p = 0.058), to highly correlated in 2013 (Philippines-and Mexico: r = 0.964, p < 0.0005; Japan and Mexico: r = 0.879, p < 0.0005). The greatest divergence in songs across the North Pacific was in 2011, and the greatest convergence in 2013, with 2012 midstream between.

### Song similarity and distance between populations

The relationship between the degree of song similarity and distance between populations was investigated through: (1) linear regression analysis of the distances apart versus percentage of shared phrases, and (2) a comparison of distance apart using the Pearson’s Correlation Coefficient values of songs.

#### Shared phrases versus geographic distance

**Figure 6:**
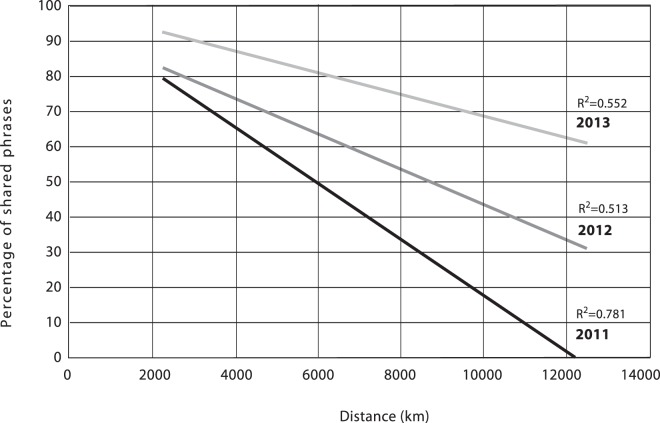
Linear regressions of percentage of shared phrases versus distance separating study locations (ranging from 2,200 to 12,000 km) for the three years, 2011, 2012, and 2013.

Linear regressions were performed to determine if there was any association of distance and percentage of shared themes for each year shown in Fig. 6. The R^2^ values were all greater than 0.50, which indicated that distance apart was indeed a factor and could explain half to over three-quarters (55.2% in 2013, 51.3% in 2012, and 78.1% in 2011) of the variation in the percentage of shared theme values. However, these values also indicate that distance apart only partially explains the data.

#### Correlation values versus geographic distance

A second view of this relationship is given in Table 4, which lists correlation values in songs for each of the three years against the distances separating them. These correlations take into consideration presence and proportion of phrase use. There is an indication of lower correlation between songs as distance increases in 2011 and even more so in 2012. This trend disappears in 2013 with high correlations between the most distant locations, in fact, higher than the closer locations in this sample (Japan-Hawaii vs. Japan-Mexico). The 2013 correlations indicate that, in that year, distance apart was not a predictor of degree of song similarity.

**Table 4:** Relationship of Pearson Correlation values and distance between song samples.

Location	Distance	Pearson’s Correlation, p-value (% shared patterns)		
	(km)	2011	2012	2013
P-J	2300	0.914*** (100)	0.958*** (71)	0.940*** (87)
H-M	4800	0.065 ns (38)	0.870*** (100)	0.852*** (100)
J-H	6200	0.673* (40)	0.853*** (50)	0.676*** (75)
P-H	8400	0.743** (40)	0.809** (30)	0.869*** (62)
J-M	10,500	−0.377 ns (10)	0.560 ns (50)	0.879*** (75)
P-M	12,500	−0.398 ns (10)	0.451 ns (30)	0.964*** (62)

Correlation is significant at 0.001 level***, 0.01 level**, 0.05 level* (two-tailed). The percentage of shared phrases between songs at different distances is also listed (in brackets). P = Philippines, J = Japan, H = Hawaii, M = Mexico.

### Within season change in 2013 Japan song

**Figure 7:**
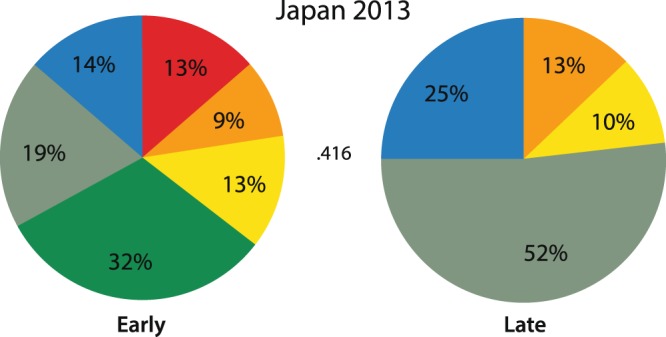
Composition of early (Jan./Feb.) and late (Mar./Apr.) song in Ogasawara, Japan 2013. The Pearson Correlation value 0.416 is not significant.

During the course of the Japan 2013 analysis, which included 12 singers recorded from January to April, a relatively greater degree of difference was noticed between early and late songs than in other samples. To examine this difference further, the season was divided into the first half’s (Jan./Feb.) six songs and second half’s (Mar/Apr.) six songs. As illustrated in Fig. 7, Phrase 1 (red), and Phrase 4a (dark green) prominent in early 2013 (and years prior) were not present in the late season sample. The Phrase 4c variant (gray-green) went from 19% to 52% of the song between early and late samples. The correlation between the first and second half of the Japan season was low (r = 0.416, p = 0.178), lower than the average of the entire year in Japan and that of the other locations in the North Pacific.

### Relative outlier 2011 Mexico song

**Figure 8:**
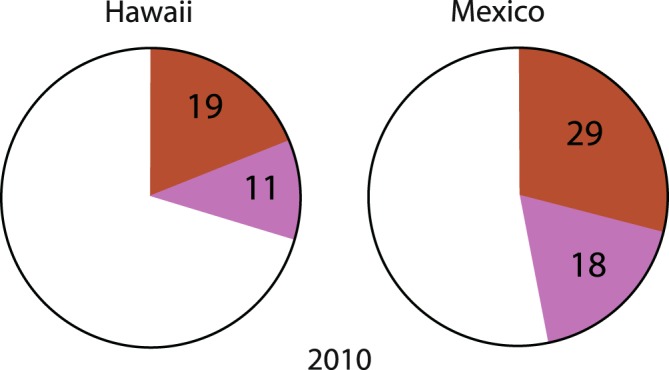
Percentage of Phrases 7 and 8 in the Hawaii and Mexico 2010 song, the year before this study.

The higher degree of uniqueness in the Mexico 2011 song relative to other years and locations led to further examination of its context. Three songs recorded in each of Hawaii and Mexico in 2010, the year previous to this study, were analyzed. As illustrated in Fig. 8, Phrases 7 and 8, the phrases largely responsible for this 2011 Mexico uniqueness, were present and prominent in both locations in 2010 and a significant part of the song prior to this study period.

## Discussion

Over at least 35 years (1977–2013) humpback whales in different North Pacific winter assemblies – in Mexico, Hawaii, Japan and Philippines − shared all, or portions, of complex, ever-changing songs^17–23^; studies listed in Table 5. The interpretation by these studies was that whales within the populations compared mixed at some point during annual cycles and in doing so, somehow, blended songs. The long-term result of this behavior has been the maintenance of a largely common, collectively changing song across the ocean basin. This study is consistent with the earlier work.

**Table 5:** Shared song phrases between populations across the North Pacific identified over the last 35 years.

Locations	Song Phrases		
	Total	Shared	% Shared
Mexico – Hawaii			
1978^17^	4	4**	100
1979^17^	4	4**	100
1977^18^	9	5	55
1979^18^	7	7	100
1989^19^	8	5	63
1991^21^	7	7	100
2011*	8	3	38
2012*	8	8	100
2013*	6	6	100
Japan – Hawaii			
1988^20^	6	5	83
1989^19^	7	4	57
2006^23^	13	9	69
2011*	7	4	57
2012*	10	4	40
2013*	8	4	50
Japan – Mexico			
1989^19^	9	3	33
2011*	8	1	13
2012*	10	4	40
2013*	8	4	50
Philippines - Hawaii			
2002^22^	9	7	77
2006^23^	13	9	69
2011*	7	3	43
2012*	9	3	33
2013*	8	5	63
Philippines - Japan			
2006^23^	9	9	100
2011*	5	5	100
2012*	7	5	71
2013*	7	6	86
Philippines – Mexico			
2011*	8	1	13
2012*	9	3	33
2013*	8	5	63

The definition of the phrases compared was not necessarily comparable author to author, year to year, however, the percentage of phrases shared is meaningful.

*This study’s results, **Authors^17^ state songs ‘virtually identical’.

However, this study showed that while the rule over time was a common ‘greater’ song across the North Pacific the percent of shared phrases between winter assemblies varied from year to year. This was immediately evident with these comparisons over three subsequent years; on review, earlier studies collectively indicate a similar pattern (Table 5). For example, one study^18^ reported Mexico and Hawaii sharing 55% of  phrases  in 1977, and 100% of phrases in 1979. It is apparent that in the North Pacific, the degree of song similarity (based on presence and proportion of different phrase types) reported between winter assemblies depends on the year the sampling was done, and may even depend on time of year, as indicated by the change in Japan 2013 song from early to late in season.

Rather than static or constant, the differences between winter assemblies may be better viewed as fluid divergences from a more common North Pacific song. While the degree of difference between assemblies *varied* from small to substantial, these states were observed to be *temporary* – and reversible. Overall, song divergences were markedly limited relative to the potential of four or more completely different songs in the North Pacific. It is apparent that over an extended time period, regardless of temporary divergences, songs change collectively across the entire North Pacific.

The question is why the degree of song similarity varied year to year between winter populations with the same geographic spacing? The most likely explanation is that this variability was a reflection of a differing degree of interaction year to year of whales from the different winter assemblies. As hypothesized, more similar song would indicate greater mixing of whales while its divergence would indicate less mixing and extended periods of isolation.

Hard evidence for this relationship is limited but developing. One study which compared Mexico and Hawaii 1999 songs reported small-scale but increasing song differences as the winter season progressed^21^. This suggests that isolation via distance led in short order towards song divergence. Conversely, song convergence was reported from Australia in 2000 when the mixing of previously isolated whales led to song conformity. In this case, whales in eastern Australia changed their song to match that of whales from western Australia, presumably due to an influx of whales from the west^4^.

According to this construct, the low correlation of song in 2011 between Asia and Mexico, and even between Hawaii and Mexico would reflect a greater degree of isolation of the Mexico population prior to that season; and the high correlation of song in 2013 across the breadth of the North Pacific would indicate far more mixing had occurred prior to that date. It is important to acknowledge here that beyond the need to be within acoustic range, the actual location and season of song adoption or blending is unknown.

The topic of the mechanism(s) of change in an individual’s and population’s song is complex and largely beyond the scope of this report, however two observations are notable. The first is that song change was not necessarily steady: characterized by periods of relative stability with spurts of greater change. This suggests that some North Pacific change is event-based, similar to when the western Australia singers rapidly changed eastern Australia song by intruding into the song pool^4^. Within this current study, the clearest potential example of an event-based change was in mid-season 2013 when the Asia song took a relatively rapid turn to greater similarity with songs from further east.

The second insight is that different populations may have different sensitivity or propensity to change, an idea that will require a longer study to confirm. In this study, Mexico appeared more ‘acoustically dynamic’ (60% song change year to year) than Hawaii (20% song change year to year). Combined, these observations suggest song change may result from the interplay of (at least) two factors: the degree of exposure to different songs providing the raw material for change; and, the sensitivity of the current song to change, speculatively, a function of the number of new songs introduced relative to the size of pool of ‘pre-existing song’ whales. For example, perhaps ‘Mexico’ whales spread further in the summer than ‘Hawaii’ whales increasing exposure to a greater variety of songs, and/or may have a more complex distribution in winter that promotes song divergences, and as well have a smaller population or pool of singers than Hawaii, making change more likely^32,33^.

A key objective of this study was to examine the relationship between the degree of song similarity and degree of association of whales − using geographic distance apart as a measure of association. The further whales are apart, the less likely they would associate and the greater the song difference. Many of the results were consistent with this model, including trends to fewer shared phrases and weaker correlation values with increasing distance, and with exclusive phrases found only in populations furthest distant from each other. Earlier multi-location, same ocean, song studies indicated similar relationships^19,23,24,28^. Critically, however, this relationship between geographic distance and mixing is not a constant, or even a given, in the North Pacific. Other factors may override it in some years, resulting in broader amalgamation and mixing of whales throughout the ocean basin. In the South Pacific song similarity also decreased as distance increased, but it was noted that, at times, adjacent populations could be connected^28^.

This study suggests that in the North Pacific song does indeed reflect humpback whale associations but these  associations are not limited by, or a function of, geography. Other, variable environmental, ecological or social factors ultimately determine the distribution of humpback whales and the clumping or splitting of subpopulations, and, in turn, affect song consistency or variety across the ocean at any given time. This raises the possibility that the song dynamic of divergence and convergence is a reflection of the group dynamics of humpback whales reacting to large-scale ecological (e.g. prey) or environmental (e.g. sea temperature) variability in the North Pacific ocean.

Extensive humpback whale song comparisons have been undertaken in the southeastern South Pacific including eastern Australia, New Caledonia, Tonga, Samoa, Cook Islands and French Polynesia^4,16,28^. However, apparent differences in song dynamics between the hemispheres complicate a comparison. These include, in the South, rapid changes in the entire song, and several entirely different songs present at any one time across the region − neither have been reported in the North^4,16^. However, a study of South Pacific humpback whale population structure based on song proposed a metapopulation structure^28^, a broad concept where spatially separated populations interact at some level, one that may also be applicable to the North Pacific^21^. The possibility of a similar population structure but differing song dynamics between hemispheres is intriguing and warrants further investigation.

This study, along with previous song comparison studies, indicates that populations of humpback whales throughout the North Pacific mix on an ongoing basis, and have done so for as long as we have studied them. Photo-identification matches and satellite tagging clearly indicate the potential for ocean-wide mixing, with individuals on one feeding ground migrating to different winter assembly locations; individuals changing their winter destination year to year - and even one record of multiple crossings of the Pacific from Asia to the North American coast^32–42^. However, to date the import of this mixing has often been minimized, as separate North Pacific ‘stocks’ were designated^29,33,40^.

The recent NOAA/NMFS (2016) regulatory action  divided the North Pacific humpback whale population into four ‘Distinct Population Segments’ (DPSs) based on winter assemblies, and gave them different status: Central American – Endangered; Mexico – Threatened; Hawaii – no protection warranted; and Western North Pacific – Endangered^43^. The NMFS decision portrays the different North Pacific populations being independent, biologically discrete and distinct (hence managed separately), whereas songs suggest they may actually be integrated, interactive and temporary – making, among other things, the different status designations questionable.

Do these large scale, ocean-wide, population-level comparisons provide insight into the biological function of the song? To date, all studies agreed that shared song composition means singers have associated, at least acoustically, in some recent time frame^3,4,11,15–19,21,23^. This study described the fluidity of song composition within and between the North Pacific groups, presumably the result of constantly changing associations, of, at least, males, that include, perhaps necessitate, real-time acoustic definition. It appears the song provides an immediate social identity to transitory groupings of whales. Whether this is a clue to its function is not known. What whales might do with such information is not known.

## Data Availability

The datasets generated during and/or analysed during the current study are available from the corresponding author on reasonable request.

## Supplementary information

Convergence and divergence of songs suggest ongoing, but annually variable, mixing of humpback whale populations throughout the North Paciific

## References

[1] Payne RS, McVay S (1971). Songs of humpback whales. Science..

[2] Darling JD, Bérubé M (2006). Interactions of singing humpback whales with other males. Mar. Mamm. Sci..

[3] Payne, K., Tyack, P. & Payne, R. S. Progressive changes in the songs of humpbacks: a detailed analysis of two seasons in Hawaii in Communication and *behavior of whales* (ed. Payne, R.) 9–57 (Westview Press, 1983).

[4] Noad MJ, Cato DH, Bryden MM, Jenner MN, Jenner KCS (2000). Cultural revolution in whale songs. Nature..

[5] Gabriele CM, Frankel AS (2002). The occurrence and significance of humpback whale songs in Glacier Bay, Southeastern Alaska. Arctic Res. U.S..

[6] Clark CW, Clapham PJ (2004). Acoustic monitoring on a humpback whale (*Megaptera novaeangliae*) feeding ground shows continual singing into late spring. Proc. R. Soc. Lond. B..

[7] Stimpert AK, Peavey LE, Friedlaender LS, Nowacek DP (2012). Humpback whale song and foraging behavior on an Antarctic feeding ground. PLoS One.

[8] Vu ET (2012). Humpback whale song occurs extensively on feeding grounds in the western North Atlantic Ocean. Aquat. Biol..

[9] Clapham PJ (1996). The social and reproductive biology of humpback whales: an ecological perspective. Mamm. Rev..

[10] Cerchio S, Jacobsen JK, Cholewiak DM, Falcone EA, Merriwether DA (2005). Paternity in humpback whales, *Megaptera novaeangliae*: assessing polygyny and skew in male reproductive success. Anim. Behav..

[11] Darling JD, Jones ME, Nicklin CP (2006). Humpback whale songs: Do they organize males during the breeding season?. Behav.

[12] Smith JN, Goldizen AW, Dunlop RA, Noad MJ (2008). Songs of male humpback whales, (*Megaptera novaeangliae*), are involved in intersexual interaction. Anim. Behav..

[13] Herman LM (2017). The multiple functions of male song within the humpback whale (*Megaptera novaeangliae*) mating system: review,. evaluation, and synthesis. Bio. Rev. Camb. Philos. Soc.

[14] Cholewiak, D. M., Cerchio, S., Jacobsen, J. K., Urbán R. J., and Clark, C. Songbird dynamics under the sea: acoustic interactions between humpback whales suggest song mediates male interactions. *R. Soc. open sci*. **5**, 171298, 10.1098/rsos (2018). 10.1098/rsos.171298 PMC5830736 29515847

[15] Payne. K, Payne RS (1985). Large scale changes over 19 years in songs of humpback whales in Bermuda. Z. Tierpsychol..

[16] Garland EC (2011). Dynamic horizontal cultural transmission of the humpback whale song at the ocean basin scale. Curr. Biol..

[17] Winn HE (1981). Songs of the humpback whale: Population comparisons. Behav. Ecol. Sociobiol..

[18] Payne, R. S. & Guinee, L. N. Humpback whale (*Megaptera novaeangliae*) songs as an indicator of ‘stocks’ in *Communication and behavior of whales* (ed. Payne, R.) 333−358 (Westview Press, 1983).

[19] Helweg DA, Herman LM, Yamamoto S, Forestell PH (1990). Comparison of songs of humpback whales (*Megaptera novaeangliae*) recorded in Japan, Hawaii and Mexico during the winter of 1989. Sci. Rep. Cetacean Res..

[20] Darling JD, Mori K (1993). Recent observations of humpback whales (*Megaptera novaeangliae*) in Japanese waters, Ogasawara and Okinawa. Can. J. Zool..

[21] Cerchio S, Jacobsen JK, Norris TF (2001). Temporal and geographical variation in songs of humpback whales, *Megaptera novaeangliae*: synchronous change in Hawaiian and Mexican breeding assemblages. Anim. Behav..

[22] Acebes JV, Darling JD, Yamaguchi M (2007). Status and distribution of humpback whales (*Megaptera novaeangliae*) in Northern Luzon, Philippines. J. Cetacean Res. Manag..

[23] Darling JD, Acebes JV, Yamaguchi M (2014). Similarity yet a range of differences between humpback whale songs recorded in the Philippines, Japan and Hawaii in 2006. Aquat. Biol..

[24] Helweg DA, Cato DH, Jenkins PF, Garrigue C, McCauley R (1998). Geographic variation in South Pacific humpback whale songs. Behav.

[25] Darling JD, Sousa-Lima RS (2005). Songs indicate interaction between humpback whale (*Megaptera novaeangliae*) populations in the western and eastern South Atlantic Ocean. Mar. Mamm. Sci..

[26] Razafindrakoto, Y., Cerchio, S., Collins, T., Rosenbaum, H., Ngouessono, S. Similarity of humpback whale song from Madagascar and Gabon indicates significant contact between South Atlantic and southwest Indian Ocean populations. IWC Scientific Committee June 2009 Madeira Portugal, Paper SC/61/SH8, **15** pp. (2009).

[27] Murray A (2012). Minimal similarity in songs suggest limited exchange between humpback whales (*Megaptera novaeangliae*) in the southern Indian Ocean. Mar. Mamm. Sci..

[28] Garland EC (2015). Population structure of humpback whales in the western and central South Pacific Ocean as determined by vocal exchange among populations. Conserv. Biol..

[29] Bettridge, S. *et al*. Status review of the humpback whale (*Megaptera novaeangliae*) under the Endangered Species Act 2015. NOAA-TM-NMFS-SWFSC-540. http://www.nmfs.noaa.gov/pr/species/Status%20Reviews/humpback_whale_sr_2015.pdf. **62** pp (2015).

[30] Urbán RJ, Aguayo AL (1987). Spatial and seasonal distribution of the humpback whale, *Megaptera novaeangliae*, in the Mexican Pacific. Mar. Mamm. Sci..

[31] Cholewiak DM, Sousa-Lima RS, Cerchio S (2013). Humpback whale song hierarchical structure: historical context and discussion of current classification issues. Mar. Mamm. Sci..

[32] Urbán RJ (2000). Migratory destinations of humpback whales wintering in the Mexican Pacific. J. Cetacean. Res. Manage..

[33] Calambokidis, J. *et al*. SPLASH: structure of populations, levels of abundance and status of humpback whales in the North Pacific. Final report for Contract AB133F-03-RP-00078, to U.S. Dept. of Comm. Western Administrative Center, Seattle, WA. http://www.cascadiaresearch.org/files/Projects/Archived_projects/SPLASH/SPLASH-contract-Report-May08.pdf (2008).

[34] Nishiwaki, M. Distribution and migration of the larger cetaceans in the North Pacific as shown by Japanese whaling results in *Whales, dolphins and porpoises* (ed. Norris, K. S.) 171−191 (University of California Press, 1966).

[35] Darling, J. D. & Jurasz, C. M. Migratory destinations of North Pacific humpback whales (*Megaptera novaeangliae*) in Communication and behavior *of whales* (ed. Payne, R.), 359−368. (Westview Press, 1983).

[36] Darling. JD, McSweeney DJ (1985). Observations on the migrations of North Pacific humpback whales (*Megaptera novaeangliae*). Can. J. Zool..

[37] Darling JD, Cerchio S (1993). Movement of a humpback whale (*Megaptera novaeangliae*) between Japan and Hawaii. Mar. Mamm. Sci..

[38] Darling JD (1996). Movement of a humpback whale from Japan to British Columbia and return. Mar. Mamm. Sci..

[39] Salden DR, Herman LM, Yamaguchi M, Sato F (1999). Multiple visits of individual humpback whales (*Megaptera novaeangliae*) between the Hawaiian and Japanese winter grounds. Can. J. Zool..

[40] Calambokidis J (2001). Movements and population structure of humpback whales in the North Pacific. Mar. Mamm. Sci..

[41] Mate BR, Mesecar R, Lagerquist B (2007). The evolution of satellite-monitored radio tags for large whales: One laboratory’s experience. Deep-Sea Res II.

[42] Titova OV (2018). Photo-identification matches of humpback whales (*Megaptera novaeangliae*) from feeding areas in Russia Far East seas and breeding grounds in the North Pacific. Mar. Mamm. Sci..

[43] Federal Register. Endangered and Threatened Species; Identification of 14 distinct populations segments of the humpback whale (*Megaptera novaeangliae*) and revision of species wide listing. NMFS NOAA 09/08/2016 Doc citation 81FR 62259, doc # 2016-21276, 62 pp. (2016).

