# Author Correction: Convergence and divergence of songs suggests ongoing, but annually variable, mixing of humpback whale populations throughout the North Pacific

**DOI:** 10.1038/s41598-019-45363-0

**Published:** 2019-07-11

**Authors:** James D. Darling, Jo Marie V. Acebes, Oscar Frey, R. Jorge Urbán, Manami Yamaguchi

**Affiliations:** 1 Whale Trust, Makawao, Hawaii 96768 United States of America; 2 BALYENA.ORG, Barangay Pangdan, 6308, Philippines and National Museum of the Philippines, Zoology Division, Manila, 1000 Philippines; 3 Deep Blue Conservancy, Puerto Vallarta, Jalisco, CP 48328 Mexico; 4 Universidad Autonoma de Baja California Sur, Departamento de Ciencias Marinas y Costeras, La Paz, Mexico; 5 Ogasawara Club, Komagari, Chichi-jima, Ogasawara, Tokyo, Japan

Correction to: *Scientific Reports* 10.1038/s41598-019-42233-7, published online 07 May 2019

The original version of this Article contained a typographical error in the spelling of the author Jo Marie V. Acebes, which was incorrectly given as Joe Marie V. Acebes. This has now been corrected in the PDF and HTML versions of the Article, and in the accompanying Supplementary Information file.

