# Author Correction: Convergence and divergence of songs suggests ongoing, but annually variable, mixing of humpback whale populations throughout the North Pacific

**DOI:** 10.1038/s41598-020-57441-9

**Published:** 2020-01-15

**Authors:** James D. Darling, Jo Marie V. Acebes, Oscar Frey, R. Jorge Urbán, Manami Yamaguchi

**Affiliations:** 1 Whale Trust, Makawao, Hawaii, 96768 United States of America; 2 BALYENA.ORG, Barangay Pangdan, 6308, Philippines and National Museum of the Philippines, Zoology Division, Manila, 1000 Philippines; 3 Deep Blue Conservancy, Puerto Vallarta, Jalisco, CP 48328 Mexico; 4 Universidad Autonoma de Baja California Sur, Departamento de Ciencias Marinas y Costeras, La Paz, Mexico; 5 Ogasawara Club, Komagari, Chichi-jima, Ogasawara, Tokyo Japan

Correction to: *Scientific Reports* 10.1038/s41598-019-42233-7, published online 07 May 2019

This Article contains a typographical error in the Introduction.

“Singing is largely but not exclusively seasonal, its occurrence increasing in fall feeding grounds, peaking during winter migrations and assembles, and decreasing in the spring^3-8^.”

should read:

“Singing is largely but not exclusively seasonal, its occurrence increasing in fall feeding grounds, peaking during winter migrations and assemblies, and decreasing in the spring^3-8^.”

In addition, this Article also contains an error in the Results section, under subheading ‘Presence of phrases’.

“In 2013, the year of most similarity, four (Phrases 3, 4, 5, 6,) of a total of eight phrases (50%) were shared in all four populations.”

should read:

“In 2013, the year of most similarity, four (Phrases 3, 4a, 4c, 6) of a total of eight phrases (50%) were shared in all four populations.”

