# Supplementary material for: Convergence and divergence of songs suggests ongoing, but annually variable, mixing of humpback whale populations throughout the North Pacific
Source: Sci Rep. 2019 May 7;9:7002. doi: 10.1038/s41598-019-42233-7 (PMC6505537; doi:10.1038/s41598-019-42233-7)
Supplement: Supplementary file 1 — Convergence and divergence of songs suggest ongoing, but annually variable, mixing of humpback whale populations throughout the North Paciific [file 41598_2019_42233_MOESM1_ESM.pdf]

SUPPLEMENTARY MATERIALS

Convergence and divergence of songs suggests ongoing, but annually variable, mixing of humpback whale populations throughout the North Pacific

James D. Darling [1*], Jo Marie V. Acebes [2], Oscar Frey [3], Jorge Urbán R.[4], and Manami Yamaguchi [5]

[1]Whale Trust, Makawao, Hawaii, 96768, United States of America
[2]BALYENA.ORG, Barangay Pangdan, 6308, Philippines and National Museum of the Philippines, Zoology Division, Manila 1000, Philippines
[3]Deep Blue Conservancy, Puerto Vallarta, Jalisco, CP 48328, Mexico
[4]Universidad Autonoma Baja California Sur, Departamento de Biologia Marina, La Paz, Mexico.
[5]Ogasawara Club, Komagari, Chichi-jima, Ogasawara, Tokyo, Japan
*corresponding. jimd367@gmail.com

Supplementary Methods – Specific Decisions in Analysis

Phrases were defined by the combination of all their attributes. Each phrase was different from another in composition and arrangement of units - including order, repetition, spacing and cadence. In a few cases similar sound units were present in more than one phrase; however, the overall composition and arrangement of the phrases made them readily distinguishable.

Each phrase and phrase variant was used as a comparison point between songs. However, in one Phrase (4a) the units were emitted 'simply', or with a variety of frequency and duration exuberances coined flourishes. The consistent appearance of these two 'styles' across the sample led to a division of this phrase into 'simple' (4a) and 'flourished' (4aF). In this analysis all the flourish variations were clumped into one category for comparison between locations/years. Simple and flourished phrases could alternate with each other within the theme.

A separate designation (alphanumeric and color) was assigned if 50% of the units in a phrase or phrase variant changed from the parent phrase.

One phrase (Phrase 4) consisted of an extended repetition of two sound units (designated H and I); see Supplementary Fig. S2 and Supplementary Table S2. While ultimately it was decided that the phrase (HI) would remain the comparison point, in some instances, especially in 2011 and 2012, the extended repetition of phrase was broken up by a consistent single unit and related spacing (HIHIHIHI **I** HIHIHIHI **I**), raising a question about a larger structural 'template' the H and I might fit into. An option was to designate the phrase (HIHIHIHI **I**), which is then repeated. While clearly a pattern in the song at times, it was not consistent over the three years and four locations (i.e., the independent **I** was not always present to separate the HIs); therefore, the decision was made to use (HI) as the Phrase 4 comparison point.

A similar larger 'template pattern' was found in two other phrases prominent in Mexico in 2011, (Phrase 7, RRRRRSS; Phrase 8, TTTTTTTUU) (See Supplementary Fig. S2 and Supplementary Table S2). However, these were different than the Phrase 4 situation (above) in that they were consistent across the sample with the two final separation units, SS or UU, prominent in every case. The entire sequence was designated the phrase, and hence these are substantially longer than other phrases in this comparison.

The majority of phrase types discussed and compared were repeated, consistent, and easily recognizable across the samples. However, a small percentage (<2%) of sounds that occurred once or only for a few seconds in some songs did not fit the defined phrase types and were designated only as 'other'. Also placed into this category was this study's single hybrid phrase, which appeared briefly in two songs in one year and one location.

Transition segments (usually less than 20 sec long) were simply divided in half for the calculation of time the singer spent on a specific phrase, i.e., half the transition time was given to the proceeding phrase and half to the following phrase.

The classification and comparison of phrase types was made by the principle investigator (JD) to ensure consistency across the sample.

Supplementary Figure S1

Examples of full recording spectrographs, identification of phrases and color bars. See Supplementary Fig S2 for phrase names. If known, down-arrow indicates singer dive, up-arrow indicates singer surfacing (all FFT 512, 50% overlap, Hamming window).

- Example 1. Mexico, 8 January 2011
- Example 2. Philippines, 7 Mar 2012
- Example 3. Hawaii, 28 March 2012

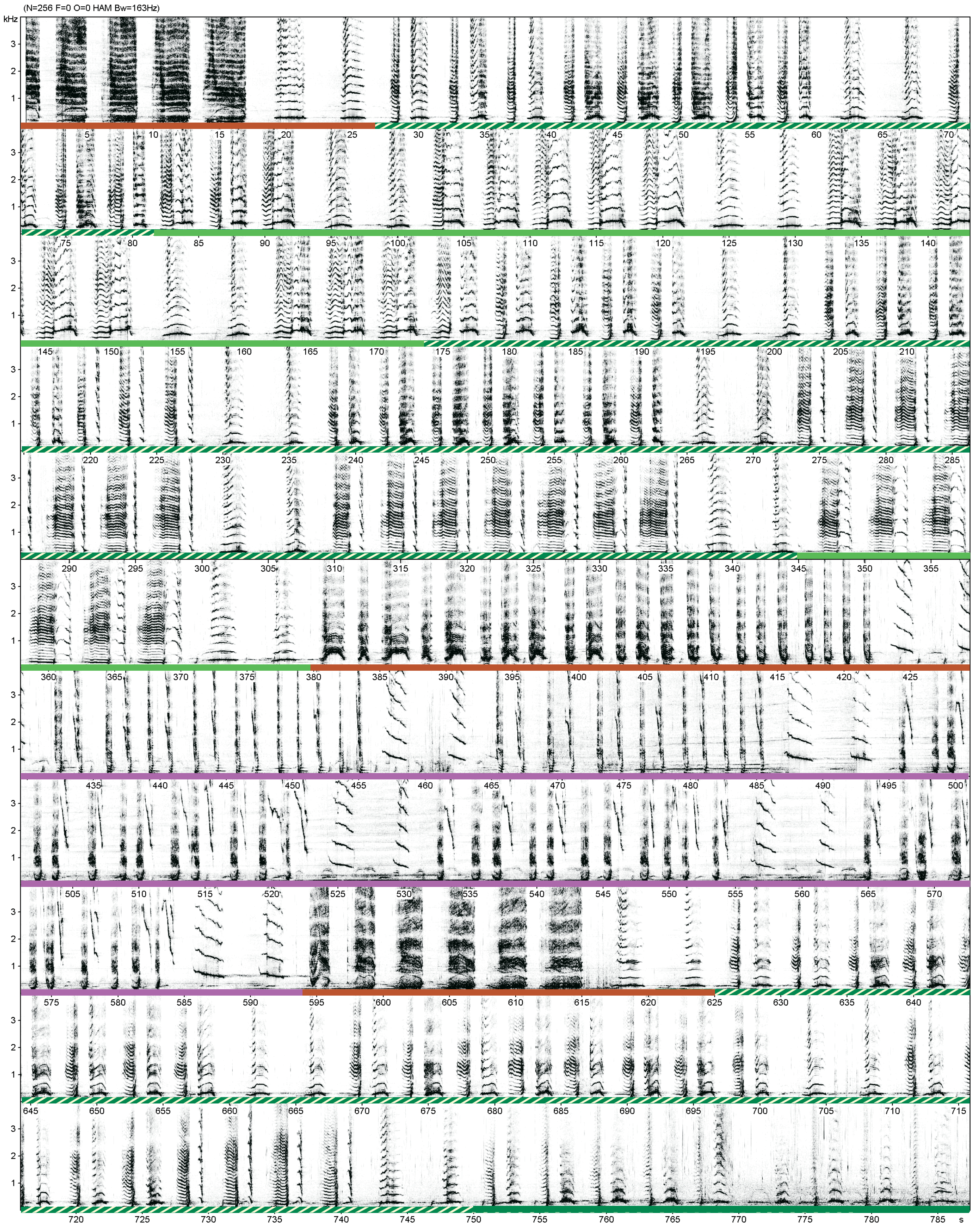

Mexico, 8 January 2011

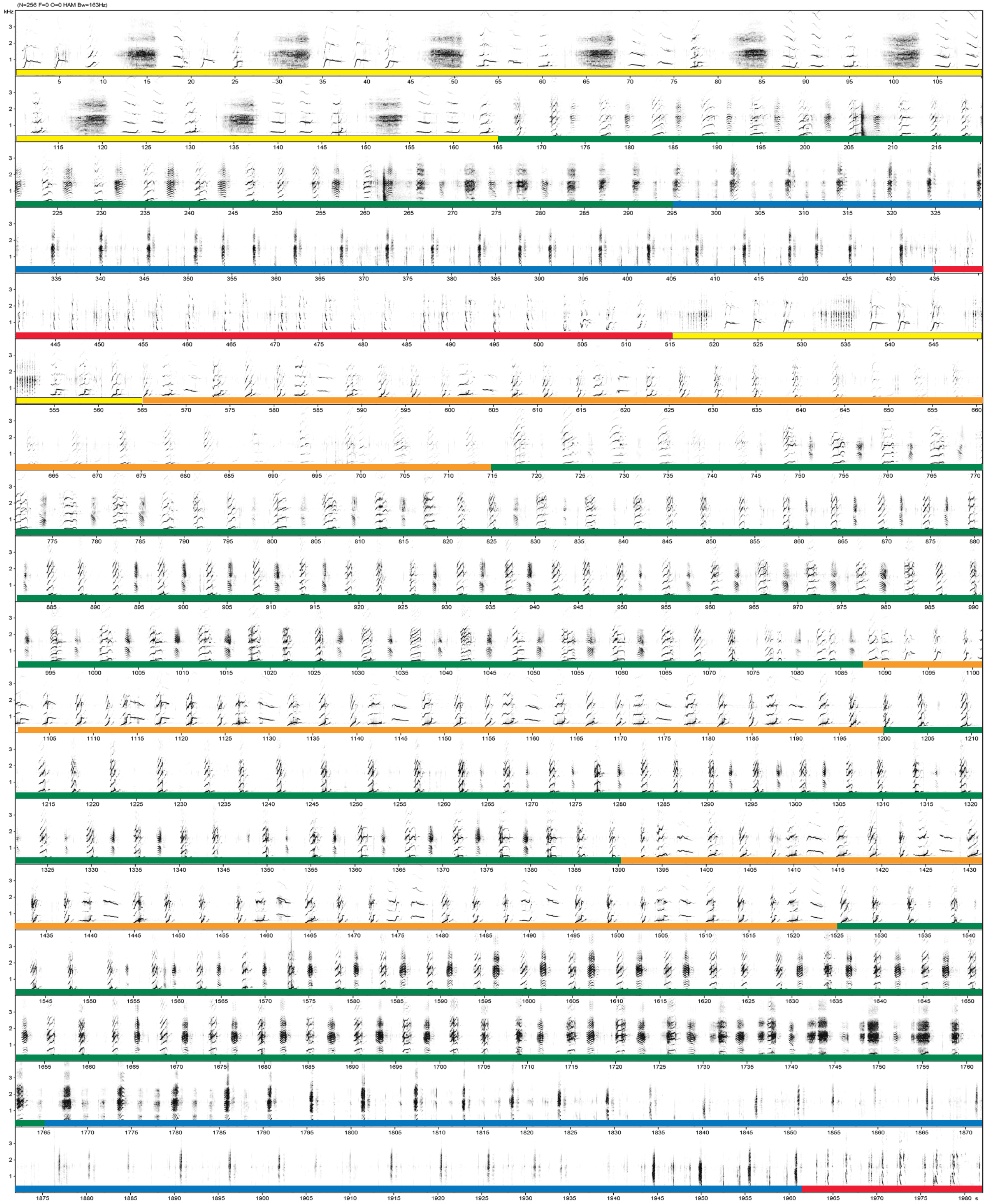

Philippines, 7 Mar 2012

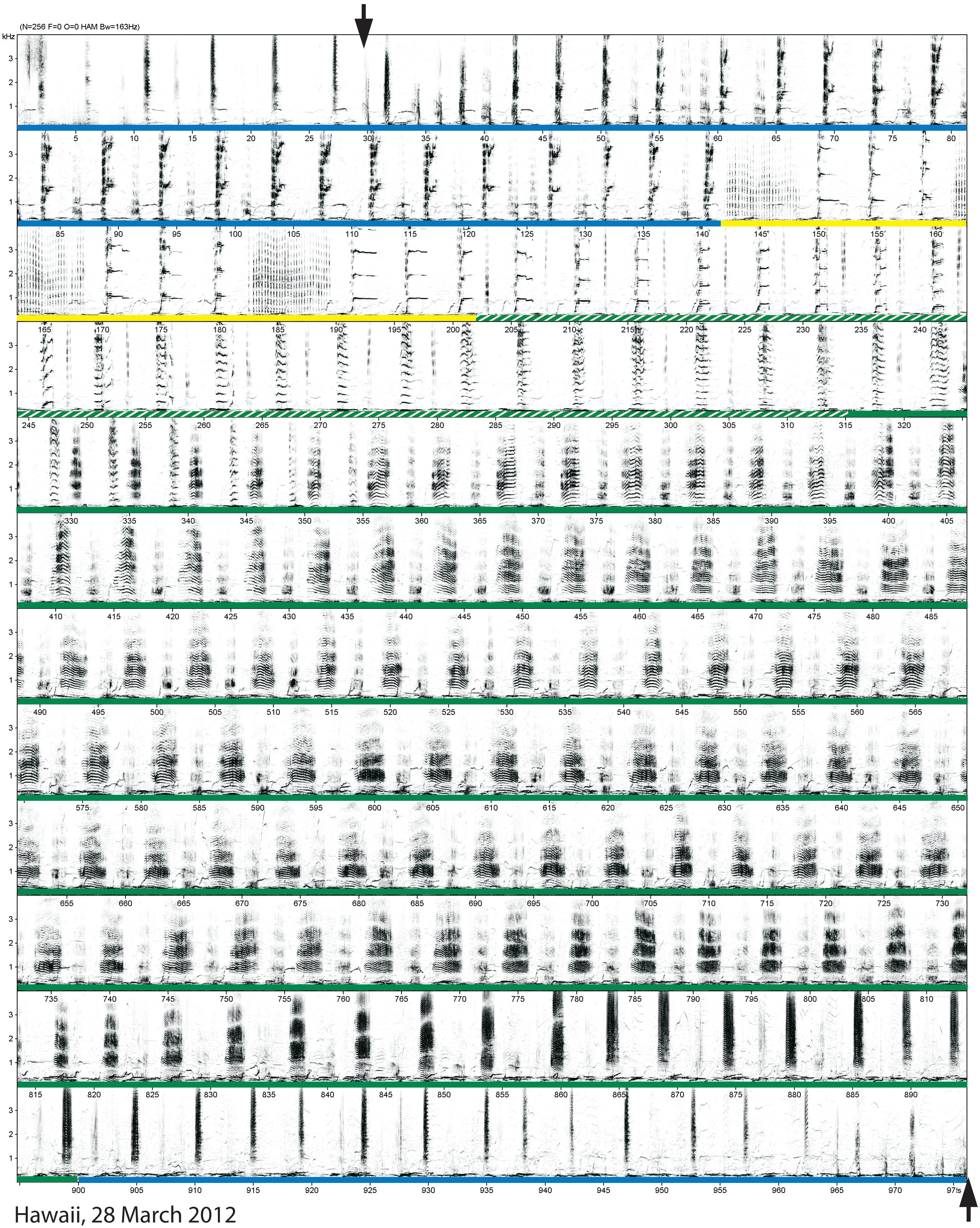

Hawaii, 28 March 2012

Supplementary Figure S2

Comparison points: spectrographs of phrases, phrase variants and phrase flourish (all FFT 512, 50% overlap, Hamming window)

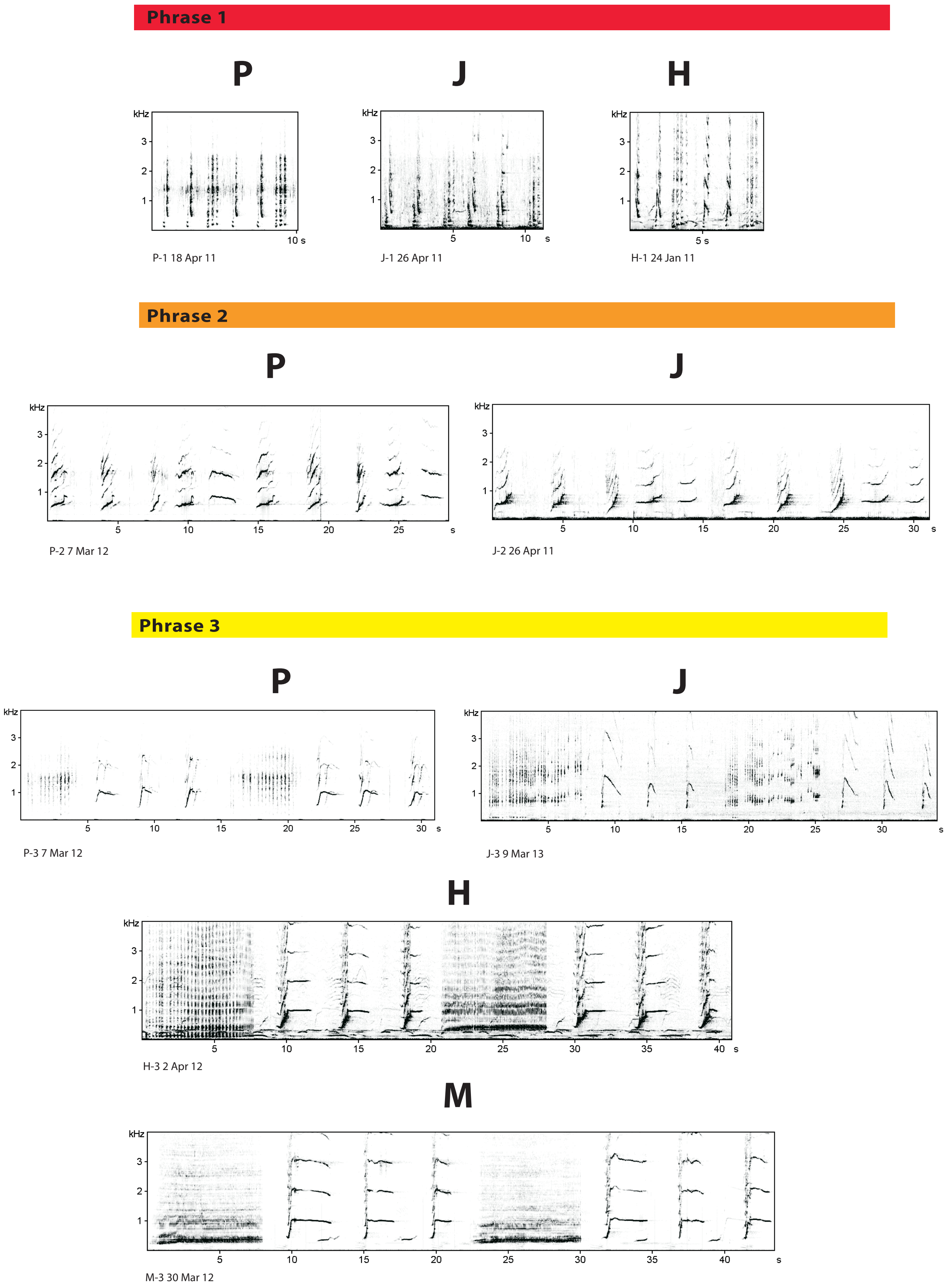

Phrase 1
P
J
H
kHz
3
2
1
10 s
P-1 18 Apr 11
5
10
s
J-1 26 Apr 11
5 s
H-1 24 Jan 11
Phrase 2
P
J
5
10
15
20
25
s
P-2 7 Mar 12
30
J-2 26 Apr 11
Phrase 3
P
J
P-3 7 Mar 12
J-3 9 Mar 13
H
35
40
H-3 2 Apr 12
M
M-3 30 Mar 12

Phrase 4a

P

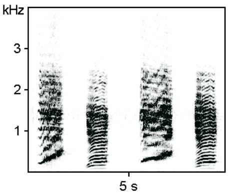

P-4a 18 Apr 11

J

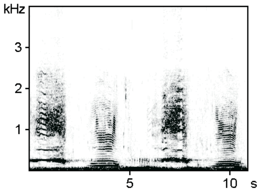

J-4a 26 Apr 11

H

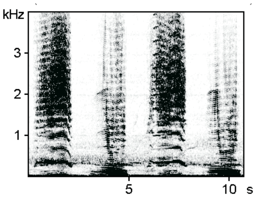

H-4a 7 Mar 11

M

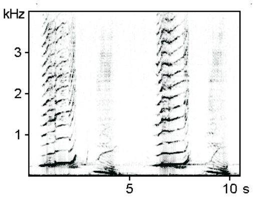

M-4a 30 Mar 12

Florish 4aF

P

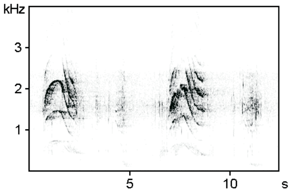

P-4aF 13 Mar 13

J

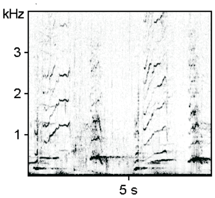

J-4aF 20 Mar 12

H

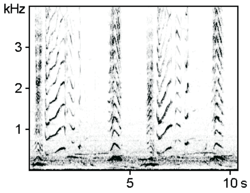

H-4aF 5 Apr 12

M

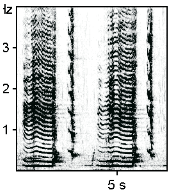

M-4aF 8 Jan 11

Variant 4b

H

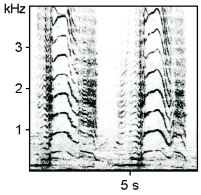

H-4b 7 Mar 11

M

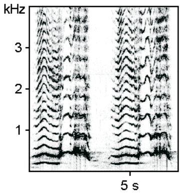

M-4b 8 Jan 11

Variant 4c

P

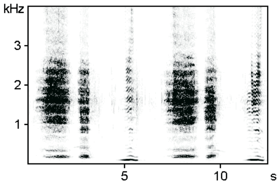

P-4c 13 Mar 13

J

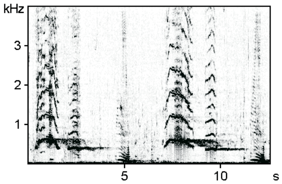

J-4c 9 Mar 13

H

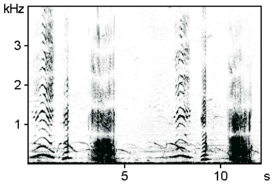

H-4c 16 Mar 13

M

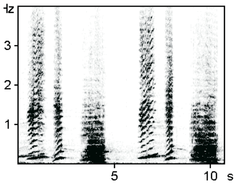

M-4c 19 Mar 13

Phrase 5

H

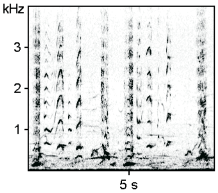

H-5 10 Jan 13

M

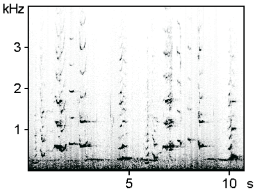

M-5 5 Feb 13

Phrase 6

P

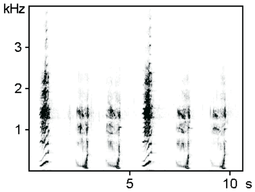

P-6 18 Apr 11

J

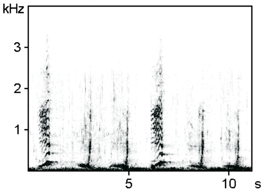

J-6 26 Apr 11

H

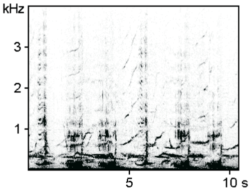

H-6 23 Jan 11

M

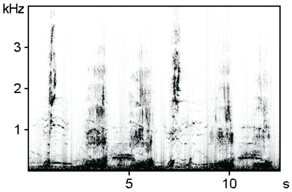

M-6 28 Feb 13

Phrase 7

M

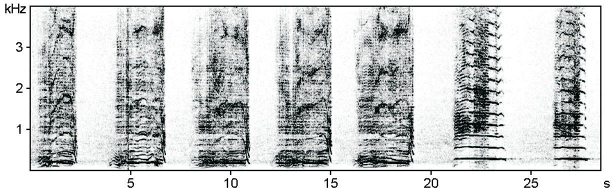

M-7 13 Mar 11

H

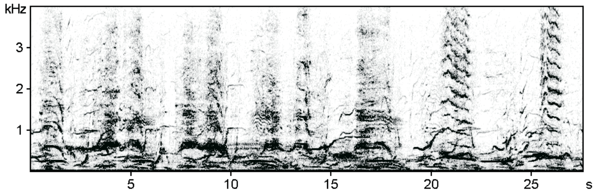

H-7 11 Jan 12

Phrase 8

M

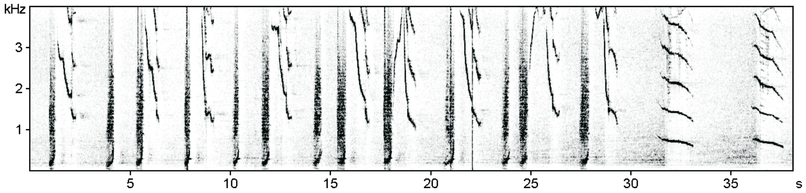

M-8 13 Mar 11

H

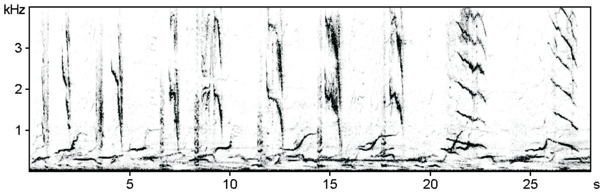

H-8 11 Jan 12

Supplementary Material Table S1 **Example of determination of percent of phrase types in a song sample**

Song sample from 16 singers in Hawaii 2013, including recording time (seconds), and calculation of percent of phrase types from, 1) proportion of total sample (bold) and, 2) mean of individual singers . Phrases 1 and 2 (in the 2011-2013 overall sample) were not present in the Hawaii 2013 sample; category of 'Other' sounds (2%) not included in this example table.

		Phrase Types											
Singer	Rec Time	3		4a		4aF		4c		5		6	
		Time	%	Time	%	Time	%	Time	%	Time	%	Time	%
1	1200	90	7.5	430	35.8	280	23.3	105	8.75	80	6.7	215	17.9
2	770	125	16.3	350	45.4	120	15.6	0	0	40	5.2	135	17.5
3	2060	35	1.7	925	44.9	5	.2	480	23.3	55	2.7	560	27.2
4	1200	130	10.8	305	25.4	40	.3	315	26.25	175	14.6	235	19.6
5	1420	115	8.1	665	46.8	45	3.2	210	14.8	130	9.15	255	17.95
6	1175	130	11.1	310	26.4	0	0	180	15.3	110	9.4	445	37.9
7	970	115	11.85	510	52.6	40	4.1	40	4.1	35	3.6	230	23.7
8	580	0	0	520	89.6	5	.9	20	3.4	0	0	35	6
9	1825	215	11.8	780	42.7	75	4.1	390	21.4	95	5.2	60	3.3
10	790	30	3.8	590	74.7	50	6.3	80	10.1	0	0	40	5
11	880	45	5.2	595	67.6	60	6.8	180	20.45	0	0	0	0
12	1050	45	4.3	410	39	80	7.6	310	29.5	40	3.8	70	6.7
13	1180	25	2.1	725	61.4	100	8.5	240	20.3	0	0	90	7.6
14	610	50	8.2	230	37.7	5	.8	175	28.7	0	0	150	24.6
15	805	50	6.2	500	62.1	105	13	120	14.9	0	0	30	3.7
16	655	0	0	285	43.5	40	6.1	300	45.8	0	0	30	4.6
Total	**17170**	**1200**		**8130**		**1050**		**3145**		**760**		**2580**	
%		**7**		**47**		**6**		**18**		**5**		**15**	
Mean SD		75 ± 58.8	6.7 ± 4.7	508.1± 197	49.7 ± 17.4	65.6 ± 68.3	6.3 ± 6.4	196.6± 138.8	17.5 ± 11.7	47.5 ± 55.6	3.8 ± 4.4	161.3 ± 158.5	14.0 ± 10.9

Supplementary Material Table S2

Pattern, duration, frequency measurements of phrases in Supplementary Figure S2

The purpose of this table is to present the arrangement, duration, spacing and frequency measurements of the phrase types to indicate: 1) the striking similarity location to location, and; 2) the range of unit variability in some phrases accepted as 'similar' in this study.

The composition of the phrases and variants were, with several minor exceptions noted below, identical between locations. If there were 21 different sound units (as letters of alphabet used in the following Table) the potential combinations are in the millions yet in this study, spanning the Pacific over three years, a total of 11 were found. The odds that the same phrase types could occur in different populations by random are negligible.

There was some minor variability in the phrase length overall between locations (see Table 3 below), and in the length and frequency contours of some units in some phrases (to the point that in Phrase 4 they were separated out as flourishes as described in the paper and in Supplementary Methods).

Minor unit variability was not of concern in this study, although fodder for further finer scale investigations. Some of this variability occurred between individual whales; some occurred within individuals with variation from one repetition of the phrase to the next (see Table 5 below). The finer the points of difference in units examined, the greater the question as to their significance. For example, a variation may be simply due to the amount of air remaining in sound-making sacs and canals.

Each table refers to the corresponding phrase spectrograph in Supplementary Fig. S2. They describe the two repetitions of each phrase (only one repetition in Phrase 7 and 8), in Fig. S2. The table includes for each location: % of the population's song the phrase occupied in each year (2011, 2012, 2013); the phrase pattern denoted by letters; the range of phrase and unit length; duration of gaps between components and; unit frequency. Notes below some of the tables draw attention to variation or other features of the phrase.

Classification and measurements were made by one person, the principal investigator (JD).

1	% Song 11,12,13	Pattern	Length (s)	Btw. phrase	UNITS										
					Unit A					Gap A-B (s)	Unit B				
					Length (s)		Frequency (Hz)				Length (s)		Frequency (Hz)		
Location					Unit	Gaps/A	Low	High	Peak		Unit	Gaps/B	Low	High	Peak
Phil.	14,6,2	AABBB	3.8-3.9	0.9	0.4	1.3	83.5-95.5	734.0-793.6	578.1-609.4	0.9-1.0	0.2	0.1	89.5-151.9	155.1-286.9	109.4-203.1
Japan	14,9,3	AABBB	4.9-5.0	0.8	0.5-0.9	1.1-1.5	60.6-90.9	1424.2-3575.8*	109.4-125.0	1.4	0.1-0.2	0.1	68.2-560.6	143.9-687.0	109.4-640.6
Hawaii	2,0,0	AABBB	3.7-3.8	1.0	0.4	1.0-1.1	93.9-381.7	903.7-1294.6	484.4-859.4	0.7-1.0	0.1-0.2	0.1	93.9-228.9	211.3-340.4	125.0-265.6
Mexico	0,0,0														

Note: Illustration of a rare pattern variation. See Fig S1, Unit B repeats 4x in one cycle of the phrase whereas 3x is most common.

2	% Song 11,12,13	Pattern	Length (s)	Btw. Phrs.(s)	UNITS										
					Unit C					Gap C-D (s)	Unit D				
					Length(s)		Frequency (Hz)				Length(s)		Frequency (Hz)		
Location					Unit	Gaps/C	Low	High	Peak		Unit	Gaps/D	Low	High	Peak
Phil.	4,8,7	CCCDE	13.2-13.3	1.5	.7-1.3	2.2-2.7	313.1-447.0	902.4-947.0	562.5-625.0	0.9-1.0	1.4-1.8	n/a	438.1-473.8	857.7-902.4	562.5
Japan	15,14,11	CCCDE	14.5-14.6	1.8	.8-1.3	2.6-3.0	175.2-437.8	852.0-932.8	617.0-695.3	1.2	1.9-2.0		528.7-579.2	862.1-902.5	609.1-625
Hawaii	0,0,0														
Mexico	0,0,0														

	Phrase 2 UNITS cont.				
	Gap D-E (s)	Unit E			
		Length	Frequency (Hz)		
		Unit	Low	High	Peak
P	1.0-1.1	1.7-1.9	563.1-723.8	902.4-982.7	781-2-843.8
J	0.8-1.0	1.4-1.6	548.9-579.2	771.2-831.8	609.4-625.0
H					
M					

3	% Song	Pattern	Length (s)	Btw. phrase	UNITS										
	11,12,13				Unit F					Gap F-G (s)	Unit G				
					Length(s)		Frequency (Hz)				Length(s)		Frequency (Hz)		
Location					Unit	Gaps/F	Low	High	Peak		Unit	Gaps/G	Low	High	Peak
Phil.	20,14,11	FGGG	13.3-14.5	2.4	4.7-5.4	n/a	1248.0-1216.0	1840.0-1968.0	1527.3-1543.0	0.6-1.0	0.8-2.0	1.2-2.5	440.8-653.1	1044.9-1142.9	753.9-1031.2
Japan	8,8,13	FGGG	15.4-15.6	2.2	7.0-7.3		576.9-615.4	2019.2-2096.2	683.6-703.1	1.3-1.4	0.6-1.6	1.3-2.1	352.9-470.6	1333.3-1705.9	539.1-1043.0
Hawaii	17,7,7	FGGG	19.1-19.3	1.3	7.3-7.4		92.0-260.5	3938.7-3954.0	105.5-418.0	1.5-1.8	1.3-2.5	1.9-2.3	321.8-398.5	980.8-1057.5	593.8-687.5
Mexico	0,11,9	FGGG	20.5-20.8	1.3	7.6-7.8		173.2-229.1	606.1-675.3	347.7-355.5	1.5	1.7-3.1	2.1-2.7	352.4-422.9	1022.0-1110.1	429.7-960.9

Note: Unit F consists of series pulses, which can be separated by ear (and spectrograph, see Fig. S1) in Philippines but less so in Japan and not at all in Hawaii and Mexico. The decision was made to treat this series as one unit, rather than 10+ separate pulses

4a	% Song	Pattern	Length (s)	Btw. phrase	UNITS										
	11,12,13				Unit H					Gap H-I (s)	Unit I				
					Length(s)		Frequency (Hz)				Length(s)		Frequency (Hz)		
Location					Unit	Gaps	Low	High	Peak		Unit	Gaps	Low	High	Peak
Phil.	39,54,29	HI	3.5-3.8	1.5	1.3-1.6	n/a	171.9-203.1	562.5-625.0	343.8-437.5	1.1-1.2	1.0-1.1	n/a	78.1-125.0	359.4-375.0	250.0-312.5
Japan	44,46,21	HI	4.0-4.1	2.1	1.3-1.5		58.0-117.6	397.1-411.8	218.8-250.0	1.4-1.5	1.1-1.2		29.4-44.1	220.6-250	31.2-62.5
Hawaii	36,48,47	HI	4.6-4.7	1.0	1.9		198.3-211.0	388.2-421.9	328.1-343.8	1.2	1.6-1.8		29.5-42.2	177.2-181.4	49.6-93.8
Mexico	4,31,28	HI	3.8-3.9	1.9	1.8-1.9		174.9-207.7	486.3-535.5	265.6	0.8	1.2		10.9-27.3	158.5-180.3	62.5

4aF	% Song	Pattern	Length (s)	Btw. phrase	UNITS										
	11,12,13				Unit H!					Gap H-I (s)	Unit I!				
					Length(s)		Frequency (Hz)				Length(s)		Frequency (Hz)		
Location					Unit	Gaps/H	Low	High	Peak		Unit	Gaps/I	Low	High	Peak
Phil.	0,0,6	H!I!	4.5-4.6	1.8	2.0-2.1	n/a	262.9-319.2	732.4-788.7	406.2-718.8	1.5-1.9	0.8-0.9	n/a	75.1	292.1-319.0	125.0-156.2
Japan	0,8,0	H!I!	3.5	1.5	0.7-0.8		73.0-119.5	212.5-225.7	171.9	1.9-2.1	0.6-0.8		272.0-278.9	464.8-484.7	406.2-437.5
Hawaii	13,27,6	H!I!	3.9-4.4	1.2	2.2-2.5		63.8-75.2	722.2-723.4	140.6	1.0-1.3	0.7		43.6-105.3	404.3-421.3	296.9
Mexico	33,41,10	H!I!	2.6-2.8	1.0	1.6-2.0		70.3-75.7	464.9-491.9	218.8-491.9	0.5-0.6	0.3-0.4		281.1-297.3	540.5	375.0-406.2

Note: This is the clumped category with just one of the flourish variations as an example. Even within this one type of flourish there is more unit variation than in other phrases, although note that the pattern, length, spacing and gaps between units are similar.

4b	% Song	Pattern	Length (s)	Btw. phrase	UNITS										
	11,12,13				Unit H-I					Gap H-I (s)	Unit n/a				
					Length(s)		Frequency (Hz)				Length(s)		Frequency (Hz)		
Location					Unit	Gaps	Low	High	Peak		Unit	Gaps/I	Low	High	Peak
Phil.	0	H-I				n/a						n/a			
Japan	0	H-I													
Hawaii	20,0,0	H-I	3.3-3.8	0.7	3.3-3.7		77.6-89.5	507.2-513.2	140.6						
Mexico	18,0,0	H-I	3.0	0.9	2.9-3.0		107.3-175.6	497.6-556.1	453.1						

4c	% Song	Pattern	Length (s)	Btw. phrase	UNITS										
	11,12,13				Unit H					Gap H-I (s)	Unit h				
					Length(s)		Frequency (Hz)				Length(s)		Frequency (Hz)		
Location					Unit	Gaps/H	Low	High	Peak		Unit	Gaps/I	Low	High	Peak
Phil.	0,0,27	HhI	5.2-5.5	1.4	1.7-2.0	n/a	91.6-106.9	251.9	171.9-187.5	0.3-0.4	0.6-0.7	n/a	99.2	244.3	171.9-187.5
Japan	0,4,33	HhI	5.6-6.0	1.5	1.4		55.3	205.8	140.6-156.2	1.5-1.6	1.3-1.6		94.9-110.7	308.3-355.7	187.5-250.0
Hawaii	0,3,18	HhI	4.3-4.4	2.8	1.1-1.2		109.8-138.6	244.0-274.5	203.1-218.8	0.3-0.4	0.5-0.6		61.0-70.6	138.6-180.4	101.6-140.6
Mexico	0,7,28	HhI	4.1-4.9	1.5	1.1-1.3		93.9-129.1	193.6-211.3	148.4-171.9	0.3	0.5-0.6		76.3-99.8	170.2-187.8	140.6-148.4

Phrase 4c UNITS cont.						
	GAP h-I (s)	Unit I				
		Length(s)		Frequency (Hz)		
		Unit	Gaps/I	Low	High	Peak
P	1.1-1.2	1.3	n/a	30.5-38.2	145	93.8
J	0.6-0.9	0.50		87.0-94.9	229.2-268.8	218.8
H	1.0	1.2		33.3	160.8-175.5	54.7-85.9
M	0.7	1.4-2.0		11.7-17.6	199.5-375.6	140.6-289.1

5	% Song	Pattern	Length	Btw.	UNITS										
	11,12,13		Full (s)	Phrase	Unit J					Gap	Unit K				
					Length(s)		Frequency (Hz)			J-K (s)	Length(s)		Frequency (Hz)		
Location					Unit	Gaps/H	Low	High	Peak		Unit	Gaps/I	Low	High	Peak
Phil.						n/a						n/a			
Japan															
Hawaii	0,2,5	JKLMO	3.8-4.2	0.7	0.4-0.6		87.7-98.7	219.3-230.2	140.6-171.9	0.1	0.9-1.0		471.4-537.2	712.6-866.1	593.8-609.4
Mexico	0,2,2	JKLMNO	4.8	0.7	0.2-0.3		124.0-157.0	231.4-247.9	171.9	0.1	1.1-1.3		297.5-314.0	619.8-628.1	546.9-593.8

Phrase 5 Units cont.												
	Gap	Unit L					Gap	Unit M				
	K-L (s)	Length (s)		Frequency (Hz)			L-M (s)	Length(s)		Frequency (Hz)		
		Unit	Gaps/L	Low	High	Peak		Unit	Gaps/M	Low	High	Peak
Phil.									n/a			
Japan												
Hawaii	0.2	0.2-0.4		559.1-570.1	789.3-877.0	671.9-812.5	0.1-0.2	0.3-0.4		241.2-592.0	405.6-734.5	343.8-687.5
Mexico	.02-0.1	0.2-0.4		578.5 603.3	743.8-760.3	640.6-718.8	0.1-0.2	0.3-0.5		652.0-669.4	710.7-801.7	593.8-742.2

Phrase 5 Units cont.												
	Gap	Unit N					Gap	Unit O				
	M-N or	Length(s)		Frequency (Hz)			N- O(s)	Length		Frequency (Hz)		
	M-O (s)	Unit	Gaps/M	Low	High	Peak		Unit	Gaps/N	Low	High	Peak
Phil.			n/a						n/a			
Japan												
Hawaii	1.0-1.2 M-O						n/a	0.6		109.6-186.4	471.4-482.4	312.5-421.9
Mexico	1.4 M-O						n/a	0.7		239.7	338.8-380.2	296.9
	0.2 M-N	0.3		528.9	752.1	585.9	1.2	0.5		239.7	380.2	296.9

Note: This phrase seemingly arose from longer units broken into a number (up to 6) of short bits with very short gaps between them. Only heard in Hawaii and Mexico songs, the pattern included in Hawaii 5 bits and in Mexico 5 bits on one repeat of the phrase and 6 on the next repeat from the same singer – an example of minor variations in pattern.

6	% Song	Pattern	Length	Btw.	UNITS											
	11,12,13		(s)	phrase	Unit P					Gap	Unit Q					
					Length(s)		Frequency (Hz)			P-Q (s)	Length(s)		Frequency (Hz)			
Location					Unit	Gaps/P	Low	High	Peak		Unit	Gaps/Q	Low	High	Peak
Phil.	22,18,18	PQQ	4.1-4.3	1.0	0.5	n/a	80.3-105.0	234.7-247.0	187.5	1.2-1.3	0.7-0.8	0.8-0.9	37.1-49.4	290.3-345.8	234.4
Japan	17,4,19	PQQ	4.4-4.5	1.1	0.6-0.7		40.2-48.2	257	140.6-218.8	1.2-1.3	0.7-1.0	0.8-0.9	24.1-56.8	257.0-266.9	62.5-109.4
Hawaii	12,11,15	PQQ	4.0-4.3	1.0	0.7-0.8		84.5-108.3	208.4-254.3	140.6	0.8-1.1	0.7 -0.8	0.8	53.5-64.5	208.4-265,2	62.5
Mexico	0,3,23	PQQ	5.2-5.5	0.7	0.4		66.9-75.3	267.8	125.0-210.9	1.7-1.9	0.9-1.1	0.9-1.1	47.3-75.3	242.6-351.5	85.9-117.2

Note: example of a stable phrase in pattern, length, spacing, unit gaps.

7	% Song	Pattern	Length	Btw.	UNITS										
	11,12,13		(s)	phrase	Unit R					Gap	Unit S				
					Length(s)		Frequency (Hz)			Q-R (s)	Length(s)		Frequency (Hz)		
Location					Unit	Gaps/R	Low	High	Peak		Unit	Gaps/S	Low	High	Peak
Phil.	0														
Japan	0														
Hawaii	0,0.5,0	RRRRRSS	26.4	n/a	1.3-2.6	1.6-2.0	377.8-404.2	701.5-766.5	546.9-640.6	1.9	2.0-2.6	2.3	248.3	453.4-496.6	267.9-312.5
Mexico	25,2,0	RRRRRSS	27.6	n/a	1.9-2.7	1.3-1.6	92.8-130.8	215.2-236.3	179.7-187.5	2.1	1.7-2.8	2.3	202.5-215.2	316.5-329.1	273.4

Note: only one phrase from each location, no repeat. The length is longer than most other phrases. Occurred only once in the Hawaii sample (in 2012); was very small part (0.50%) of Hawaii population's song.

8	% Song	Pattern	Length (s)	Btw. phrase	UNITS										
	11,12,13				Unit T					Gap T-t(s)	Unit t				
					Length(s)		Frequency (Hz)				Length(s)		Frequency (Hz)		
Location					Unit	Gaps/T	Low	High	Peak		Unit	Gaps /t	Low	High	Peak
Phil.															
Japan															
Hawaii	0,0.5,0	TTTTTT TUU	26.9	n/a	1.1-1.5	0.5-1.8	54.5-89.9	2114.1-3117.1	1554.7-2834.8		n/a	n/a	n/a	n/a	n/a
Mexico	20,2,0	TtTTtTtT TTtTTUU	36.7	n/a	1.2-2.1	0.7-1.2	18.3-28.0	3208.0-4308.6	1362-3574.5	1.0-1.4 0.5-1.0	0.3-0.5	n/a	34-45.9	439.0-609.8	193.8-279.9

Phrase 8 UNITS cont.						
	Gap T-U (s)	Unit U				
		Length(s)		Frequency (Hz)		
		Unit	Gaps/U	Low	High	Peak
P						
J						
H	2.2	2.1-2.2	2.3	324.3-432.4	792.8	617.2-632.8
M	2.1	1.4-1.7	3.0	499.9	862.1-896.6	732.1-737.5

Note: This comparison is complicated by the Mexico phrase having had 'half-units' (t), mixed in with the full units (T). The full unit consisted of a whoop attached to whistle, the half-units just the whoop (see Fig. S2 Phrase 8). The Hawaii sample just had the full units (T). Both Hawaii and Mexico examples here are just one phrase from each location. In Hawaii, similar to Phrase 7, it occurred only once in 3 years (2012) and then only in 0.50% of population's song. This is an example of a small difference in the pattern between locations; we do not have the sample size to explore this. The single occurrence of this Phrase in Hawaii was in 2012, whereas the Mexico example is from 2011. However, the overall pattern and components of the phrase are clearly similar between the locations (see Fig S2).

Supplementary Material Table S3

Pearson's Correlation Values between songs in all locations and years

Correlation is significant at 0.001 level ***, 0.01 level **, 0.05 level * (two-tailed)

Correlation Between Years Same Location			
Philippines	Pearson Correlation	p-value	Significance
2011 to 2012	0.940	< 0.0005	***
2012 to 2013	0.690	0.013	*
2011 to 2013	0.644	0.024	*
Japan			
2011 to 2012	0.960	< 0.0005	***
2012 to 2013	0.482	0.112	ns
2011 to 2013	0.454	0.139	ns
2013 early vs. late	0.416	0.178	ns
Hawaii			
2011 to 2012	0.800	0.002	**
2012 to 2013	0.846	0.001	***
2011 to 2013	0.714	0.009	**
Mexico			
2011 to 2012	0.407	0.189	ns
2012 to 2013	0.477	0.117	ns
2011 to 2013	-0.255	0.423	ns

Correlations Between Locations			
	Pearson Correlation	p-value	Significance
2011			
Philippines–Japan	0.914	< 0.0005	***
Philippines–Hawaii	0.743	0.006	**
Philippines–Mexico	-0.398	0.200	ns
Japan–Hawaii	0.673	0.016	*
Japan–Mexico	-0.377	0.228	ns
Hawaii–Mexico	0.065	0.842	ns
2012			
Philippines–Japan	0.958	< 0.0005	***

Philippines–Hawaii	0.809	0.001	**
Philippines–Mexico	0.451	0.141	ns
Japan–Hawaii	0.853	< 0.0005	***
Japan–Mexico	0.560	0.058	ns
Hawaii–Mexico	0.870	< 0.0005	***
2013			
Philippines–Japan	0.940	< 0.0005	***
Philippines–Hawaii	0.869	< 0.0005	***
Philippines–Mexico	0.964	< 0.0005	***
Japan–Hawaii	0.676	0.016	*
Japan–Mexico	0.879	< 0.0005	***
Hawaii–Mexico	0.852	< 0.0005	***
